# Discovery of a Potent Small Molecule Antagonist of GRPR for the Treatment of Pruritus

**DOI:** 10.1007/s00044-026-03566-x

**Published:** 2026-05-26

**Authors:** Mingzhou Zhou, Roland E Dolle, Amruta R Poreddy, Edmund C Hudson, Michelle A Schmidt, Margaret L Grapperhaus, Tom Gordon, Huaping Chen, Mike Prinsen, Xianyu Liu, Zhiyong E. Tao, Jinbin Xu, Ma Xenia G Ilagan, Zhou-Feng Chen

**Affiliations:** 1 Center for Drug Discovery, Washington University School of Medicine, St. Louis, MO 63110 USA; 2 Department of Biochemistry and Molecular Biophysics, Washington University School of Medicine, St. Louis, MO 63110 USA; 3 Mallinckrodt Pharmaceuticals, St. Louis, MO 63110 USA; 4 Mallinckrodt Institution of Radiology, Washington University School of Medicine, St. Louis, MO 63110 USA; 5 Center for the Study of Itch & Sensory Disorders, Washington University School of Medicine, St. Louis, MO 63110 USA; 6 Department of Anesthesiology, Washington University School of Medicine, St. Louis, MO 63110 USA

**Keywords:** Pruritus, itch, gastrin-releasing peptide receptor, GRPR, antagonist, structure-activity relationship

## Abstract

Persistent pruritus (itch) affects the quality of life, and many itch syndromes remain untreatable. Previously, we identified the gastrin-releasing peptide receptor (GRPR) playing a critical role in mediating histamine-independent itch signaling. In this study, we aimed to identify small-molecule GRPR antagonists to treat histamine-independent itch. Starting with the known small-molecule GRPR antagonist PD 176,252, we conducted systematical structure-activity relationship studies. This effort led to the discovery of antagonists that exhibit up to an 11-fold increase in binding affinity compared to PD 176,252. Compound **45** (MP-4222), a GRPR antagonist with a four-fold increased binding affinity and equal selectivity over the neuromedin B receptor (NMBR), significantly inhibited itch behavior in the chloroquine (CQ)-induced acute itch mouse model. Additionally, we attempted to reconcile the discrepancy between our in vitro experimental data and the computational docking outcome using the recently reported GRPR-PD176252 cryo-EM structure. Our studies also suggested several potential modifications to improve potency, as well as identified functional groups that have the potential in radiolabeling for targeted GRPR imaging.

**Supplementary Information:**

The online version contains supplementary material available at 10.1007/s00044-026-03566-x.

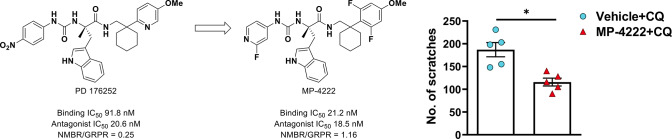

## Introduction

Itch can be a symptom of many diseases, including atopic dermatitis, contact dermatitis, psoriasis, opioid use, cholestatic liver disease, etc. [1]. It affects > 15% of the U.S. population and negatively impacts the patients’ quality of life (QoL) [2]. Itch is manifested through several signaling pathways, many of which are not fully understood [3]. The direct targeting of an itch signaling mechanism to treat itch has been very challenging. To date, only one mechanism-based anti-itch drug, difelikefalin (Korsuva), has been marketed [3]. However, difelikefalin was approved only for a small patient population: chronic kidney disease (CKD) patients who are on hemodialysis. Additionally, due to its peptidic nature, difelikefalin requires IV administration [3]. Hence, the medical needs for anti-itch treatment remain significant.

Histamine-independent itch is a common symptom for patients with opioid use and cholestatic liver disease [4]. Unlike histamine-dependent itch, which can generally be treated with antihistamine medications, histamine-independent itch has very limited treatment options [4]. We previously identified gastrin-releasing peptide receptor (GRPR) as a central neuro-signaling mediator for histamine-independent itch [5]. We demonstrated that conditional GRPR knockout mice were insensitive to the stimulation of histamine-independent itch [5]. We also found that peptide-based GRPR antagonists, such JMV641 [6], could significantly reduce mouse itch behavior through intrathecal (IT) administration in our mouse model (data not shown). However, only a handful of small-molecule GRPR antagonists have been reported [7, 8], and they demonstrated limited efficacy in our mouse models (data not shown). In this study, we describe our efforts to develop potent small-molecule GRPR antagonists with physicochemical properties suitable for in vivo efficacy studies.

## Results and discussion

### PD 176,252 benchmarking

**Fig. 1:**
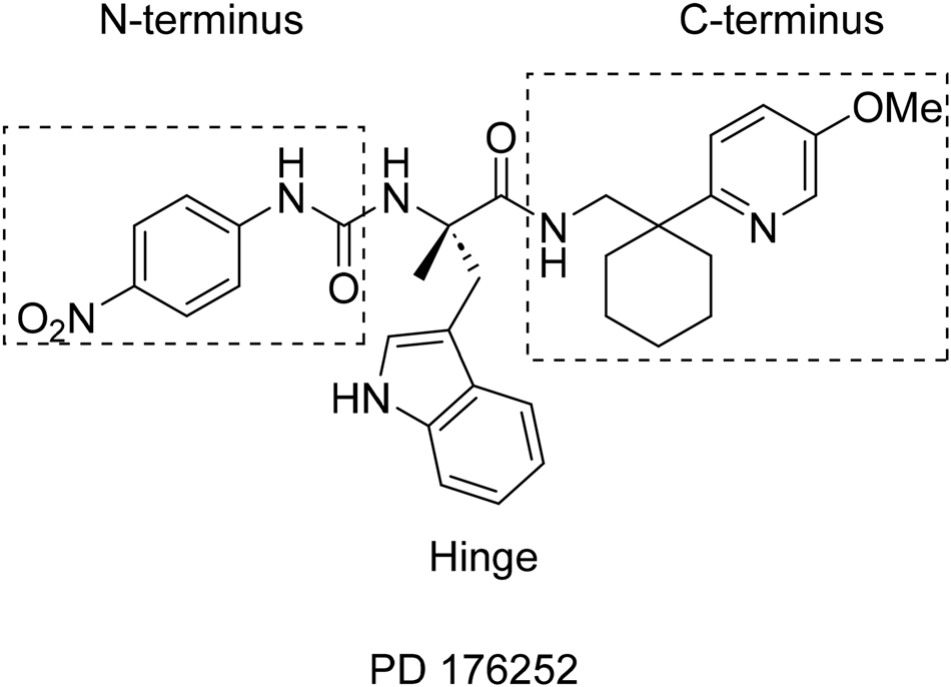
SAR development strategy of PD 176,252’s regions

PD 176,252 (Fig. 1) is one of the most potent small-molecule GRPR antagonists reported to date, being initially discovered by Parke-Davis as an NMBR antagonist. In our radiobinding assay, PD 176,252 demonstrated binding IC_50_ of 91.8 nM (Table 1). Several publications have reported attempts to improve PD 176,252’s GRPR binding affinity; however, none resulted in a more potent compound [9, 10]. In this study, our primary goal was to enhance the antagonist’s binding affinity. PD 176,252 was reported to be seven-fold more potent towards neuromedin B receptor (NMBR) compared to GRPR in the radiobinding assay [11], and in our IP-One functional assay, it was also four-fold more potent for NMBR over GRPR (Table 1). We also aimed to improve the GRPR/NMBR selectivity to reduce potential off-target side effects of inhibiting NMBR. Physicochemical properties directly impact a compound’s in vivo exposure and thereby its efficacy. While aiming to improve the potency and specificity of PD 176,252, we also sought to enhance its physicochemical properties. We benchmarked DMPK profiles for PD 176,252 (Table 2). It had low aqueous solubility; we had to apply extensive sonication in a solvent system containing 30% of 2-hydroxylpropyl-β-cyclodextrin to solubilize PD 176,252 for our in vivo itch mouse model studies. We planned to replace its nitro groups and urea groups to improve the aqueous solubility. PD 176,252 was also labile to liver microsomal metabolism and strongly inhibited multiple P450 enzymes. Additionally, it also had a short half-life in mice, and no oral bioavailability.

**Table 1:** In vitro potency of PD 176,252

	Binding IC_50_ (nM)	Functional IC_50_ (nM)	NMBR/GRPR selectivity*
PD 176,252	91.8	20.6	0.25

*By IP-one functional assay

**Table 2:** Physicochemical and preclinical pharmacokinetic properties of PD 176,252 and **45**

solubility (PBS)		PD 176,252	45
		< 1.56 µM	< 1.56 µM
Liver microsomal T_1/2_ (min)	(Human)	5.3	2.4
	(Mouse)	0.6	0.8
	(Rat)	1.8	8
% free (mouse)		99.96%	99.99%
mouse PK (T_1/2_, F)		0.57 h, 0%	ND
hERG binding IC_50_		741.6 nM	1,706 nM
CYP inhibition > 50% @ 10 µM		2C9, 2C19, 2D6, 3 A	2C9, 2C19, 2D6, 3 A

To identify the potential microsomal metabolic hot spots on PD 176,252, we conducted a metabolite identification (Met ID) study using mouse hepatocytes. PD 176,252’s primary hepatic metabolic pathways involved hydroxylations of the cyclohexyl group and the pyridine group at the C-terminus (Fig. 1). Metabolites with up to 3x hydroxylation modifications can be detected on the fragment containing the cyclohexyl group and the pyridine group. Interestingly, a common metabolic pathway, the reduction of NO_2_ to NH_2_ [12], accounted for less than 1% of the metabolism in mouse microsomes. Therefore, during the SAR studies, we planned to identify bioisosteres for the metabolically susceptible cyclohexyl and pyridine groups.

### In vitro assay to assess potency

A radiobinding assay was used as the primary assay to assess compounds’ binding affinity to GRPR. The assay was adapted from a procedure described previously [13]. In brief, we evaluated our compounds’ ability to displace the ^125^I-labeled natural ligand of GRPR, bombesin. The radiobinding assay was conducted in a two-step approach. Initially, we determined the percentage competitive displacement of new analogs against bombesin-Tyr-[^125^I] at [100 nM] and [1,000 nM]. Compounds that demonstrated > 50% displacement at [100 nM] were then subjected to a full dose-response curve to determine their binding IC_50_s.

Additionally, we established a functional assay based on the Cisbio HTRF IP-One assay. This assay measures the accumulation of IP1, a downstream metabolite produced after agonistic stimulation of Gq-coupled receptors such as GRPR. Compounds with binding IC_50_s < 100 nM were further analyzed in the GRPR IP-One assay to determine their functional IC_50_s.

## Structure-activity relationship (SAR)

To facilitate the SAR development, we investigated three structural regions of the lead: the N-terminus, the hinge and the C-terminus (Fig. 1). We started by modifying each region independently and then combined the best modifications, leading to **45**.

We began our lead optimization from the N-terminus (Table 3). Nitro substitution was generally unfavored in drug molecules due to in vivo safety concerns [12] and it is also known to reduce solubility. We sought to identify replacements for the nitro group in PD 176,252. When we replaced the N-terminal 4-nitrophenyl group with 2-, 3- and 4-pyridyl group (Tables 1 and 2–4), we found that the 4-pyridyl group (**4**) improved binding affinity and retained most of the antagonistic potency. We then tested different substitutions at this 2-pyridyl position (**5**–**7**) and observed further improvements in binding affinity of up to four-fold.

**Table 3:** Binding affinity and antagonistic activities of **1–13**


	*R*	Radio-binding		IP-One
		[100 nM] (%displacement)	IC_50_ (nM)	IC_50_ (nM)^a^
**1** **PD 176,252**		N.D.^b^	91.8	20.6
**2**		10%	N.D.	N.D.
**3**		1%	N.D.	N.D.
**4**		60%	78.6	N.D.
**5**		91%	41.9	31.4
**6**		77%	10.5	30.5
**7**		82%	29.2	116.8
**8**		67%	67.5	N.D.
**9**		-13%	N.D.	N.D.
**10**		29%	N.D.	N.D.
**11**		17%	N.D.	N.D.
**12**		82%	23.6	48.1
**13**		36%	N.D.	N.D.

^a^ Normalized to the IC_50_ of JMV-641; ^b^ not determined

The pKa of the N-terminal pyridine group significantly influenced potency. A key difference between **6** and **7** was the pKa of their pyridine group, with **6** being almost four-fold more potent. The same observation applied to **4** and **5**. Interestingly, although **5** had a binding affinity that is four-fold weaker than **4**, their antagonist potencies in the IP-One functional assay were equivalent. This indicated a disconnect between binding affinity and antagonistic effects at this binding pocket.

The position of the nitrogen atom on the aromatic ring affected binding affinity, with the *para*-position being optimal. Nitrogen substitutions at other positions (**2**, **3**, **9**, and **10**) resulted in a loss of binding affinity. We also tested the nitro group bioisosteres trifluoromethyl (**11**) and lactone (**12**) [14]. The lactone exhibited a four-fold increase in binding affinity but a two-fold loss in antagonistic potency compared to PD 176,252. Extending the N-terminus with one extra carbon also decreased the binding affinity (**13**).

To summarize, we discovered that both the 2-substituted 4-pyridyl group and isobenzofuzanone may substitute for the N-terminal nitrophenyl group of PD 176,252. These modifications improved overall binding affinity by nine-fold with similar functional antagonism.

We next investigated the SAR of the urea group (Table 4). The urea group has a relatively large PSA and two hydrogen-bond donors, which could affect permeability. Replacing the left-hand side NH of the urea group with CH_2_ resulted in a complete loss of binding affinity (**14** and **15**). We also tested if the urea group can be fused with the N-terminal aromatic ring (**16** to **27**). Of all these compounds, **19** and **24** demonstrated binding IC_50_s < 100 nM. Interestingly, **19** is a close analog of **4**, and **24** is a close analog of **1** (PD 176252). This outcome aligned with our SAR studies for the nitrobenzene group. Nevertheless, **19** and **24** were less potent compared to their urea analog. In summary, our experimental data indicated that the N-terminal urea group is involved in strong binding interactions and is challenging to replace.

**Table 4:** N-terminus and urea modifications. Binding affinity and antagonistic activities of **14–27**


	*R*	Radio-binding		IP-One
		[100 nM] (%displacement)	IC_50_ (nM)	IC_50_ (nM)^a^
**14**		2%	N.D.^b^	N.D.
**15**		-15%	N.D.	N.D.
**16**		13%	N.D.	N.D.
**17**		-25%	N.D.	N.D.
**18**		-33%	N.D.	N.D.
**19**		66%	107.4	N.D.
**20**		29%	N.D.	N.D.
**21**		-34%	N.D.	N.D.
**22**		30%	N.D.	N.D.
**23**		6%	N.D.	N.D.
**24**		70%	N.D.	N.D.
**25**		17%	N.D.	N.D.
**26**		18%	N.D.	N.D.
**27**		36%	N.D.	N.D.

^a^ Normalized to the IC_50_ of JMV-641; ^b^ not determined

After studying the SAR of the N-terminus, we tested several α-methyl noncanonical amino acids (**28** to **33**) to determine if the hinge α-methyltryptophan moiety could be replaced (Table 5). Unfortunately, none of the new analogs demonstrated binding affinity < 1 µM, including a close analog, benzofuran derivative (**28**). In the end, we decided to retain this hinge α-methyl tryptophan moiety.

**Table 5:** Hinge modifications. Binding affinity and antagonistic activities of **28–33**


	*R*	Radio-binding		IP-One
		[100 nM] (%displacement)	IC_50_ (nM)	IC_50_ (nM)^a^
**28**		8%	N.D.^b^	N.D.
**29**		31%	N.D.	N.D.
**30**		-12%	N.D.	N.D.
**31**		3%	N.D.	N.D.
**32**		14%	N.D.	N.D.
**33**		4%	N.D.	N.D.

^a^ Normalized to the IC_50_ of JMV-641; ^b^ not determined

A Met ID study revealed that the C-terminal cyclohexyl group is prone to liver metabolism; therefore, we attempted to replace or stabilize this group (Table 6). Removal of this cyclohexyl group resulted in a complete loss of binding affinity (**34**), and even reducing the ring size from six-membered to five-membered led to loss of binding affinity (**35**). Surprisingly, replacing the ring with tetrahydropyran (**36**) or substituting the C4 position using difluoride (**37**) also resulted in a significant loss of binding affinity. Furthermore, hydrophilic groups (**38** and **39**) were incompatible with this binding pocket. All the data strongly suggested that the cyclohexyl group cannot tolerate any modifications. We also attempted to restrict the flexible C-terminus by fusing an additional ring (**40**). This also resulted in a significant loss of binding affinity.

**Table 6:** Cyclohexyl modifications. Binding affinity and antagonistic activities of **34–40**


	*R*_1_, *R*_2_	Radio-binding		IP-One
		[100 nM] (%displacement)	IC_50_ (nM)	IC_50_ (nM)^a^
**34**		25%	N.D.^b^	N.D.
**35**		52%	N.D.	N.D.
**36**		37%	N.D.	366
**37**		6%	N.D.	N.D.
**38**		33%	N.D.	N.D.
**39**		6%	N.D.	N.D.
**40**		59%	242	332

^a^ Normalized to the IC_50_ of JMV-641; ^b^ not determined

Next, we studied the SAR of the C-terminus 6-(3-methoxy)pyridyl group (Table 7). Either removing the nitrogen atom from the pyridine ring (**41**) or adding an additional nitrogen atom to form a pyridazine ring (**42**) resulted in a significant reduction of binding affinity. Shifting the 3-methoxy group to the 4-methoxy position (**43**) also led to a reduction of binding affinity. We also explored substituting the pyridine group with fluoro-substituted phenyl groups. While monofluorophenyl replacement (**44**) resulted in a reduction of binding affinity, we were pleased to find that replacing the pyridine using meta-difluorophenyl (**45** and **46**) improved binding affinity 4-fold and 11-fold, respectively, with similar functional potencies. Compound **46** gave a single digit nanomolar binding affinity. To the best of our knowledge, it is one of the small-molecule GRPR binders with the greatest binding affinity.

**Table 7:** C-terminus modifications. Binding affinity and antagonistic activities of **41–49**


	*R*_1_	*R*_2_	Radio-binding		IP-One		
			[100 nM] (%displacement)	IC_50_ (nM)		IC_50_ (nM)^a^	
**41**			18%	N.D.^b^		N.D.	
**42**	“		33%	N.D.		N.D.	
**43**	“		10%	N.D.		N.D.	
**44**			39%	N.D.		N.D.	
**45** **(MP-4222)**	“		66%	21.2		18.5	
**46**		“	75%	8.1		22.9	
**47**			52%	N.D.		N.D.	
**48**			14%	N.D.		183	
**49**	“		-7%	N.D.		N.D.	

^a^ Normalized to the IC_50_ of JMV-641; ^b^ not determined

Previous studies have shown that the methoxy group at the C-terminus is crucial for antagonist potency [9]; therefore, we were surprised to find that an oxazole group (**47**) at this position could achieve < 100 nM binding affinity. However, this appeared to be unique to the oxazole group, as replacing it with either imidazole (**48**) or N-methyl imidazole (**49**) resulted in a significant reduction of binding affinity.

PD 176,252 has a peptidomimetic structure. During the SAR studies, we observed that PD 176,252 contained a tryptophan residue, which was also present in all peptide-based GRPR antagonists, such as bombesin and JMV-641. Indole is a privileged binding group [15]; therefore, we speculated that the tryptophan side chains might bind to the same pocket. Additionally, many of the reported peptide antagonists have a D-Phe or D-pentafluoroPhe at the bombesin-6 position [16], prompting us to investigate if our antagonists could access the pocket for the D-Phe side chains (Table 8). Interestingly, **52** and **53**, which had a phenyl group at the opposite side of the N-terminal isobenzofuranone group, demonstrated similar affinity and potency. After the cryo-EM structure of GRPR was published, we found that PD 176,252 and bombesin(6–14) did not share the binding pocket, and their tryptophan residues bind to different parts of GRPR. Docking studies also revealed that the phenyl groups of **52** and **53** were solvent exposed, making them suitable candidates for radio-labeling for GRPR imaging [17].

**Table 8:** N-terminus modifications. Binding affinity and antagonistic activities of **50–54**


	*R*	Radio-binding		IP-One
		[100 nM] (%displacement)	IC_50_ (nM)	IC_50_ (nM)^a^
**50**		7%	N.D.^b^	N.D.
**51**	^*^ Absolute chirality undetermined	44%	N.D.	N.D.
**52**		18%	N.D.	N.D.
**53**	^*^ Absolute chirality undetermined	70%	46.3	22.4
**54**		63%	56.8	58.1

^a^ Normalized to the IC_50_ of JMV-641; ^b^ not determined

Overall, through SAR studies, we identified a new small-molecule GRPR antagonist lead, **45** (MP-4222), which exhibits greater affinity and greater antagonistic functional potency than PD 176,252.

## Comparison between experimental binding potency and computational docking outcomes

We conducted computational docking experiments of our compounds using the GRPR-PD 176,252 structure (PDB: 7W41) and compared the docking scores with our experimental binding affinity data. In general, the docking scores and the experimental binding affinity data did not correlate well.

The cryo-EM structure explained why the urea group at the N-terminus region was essential as both of its hydrogen atoms acted as hydrogen bond donors. Additionally, the cryo-EM structure helped to elucidate why **52** and **53** exhibited similar binding affinity: both of their phenyl groups could form strong interactions with the flexible Phe178 of GRPR. This additional interaction might also explain the observed potency increase for both compounds. Conversely, the docking scores for **2**, **3**, **4**, **9** and **10** were similar, despite these compounds showing greater than ten-fold difference in their binding IC_50_s. This discrepancy could be due to the docking experiment identifying no interactions between the pyridine nitrogen of the antagonists and GRPR.

The largest discrepancy between modeling and our experimental data lay in the cyclohexyl group of the C-terminus. Our IC_50_ data demonstrated a very tight SAR at the cyclohexyl group, which the docking experiment did not capture. Compounds **34** to **39** had similar binding scores as PD 176,252, despite most of these compounds being inactive. The cryo-EM structure did not show a well-defined pocket on GRPR to accommodate this cyclohexyl group. In contrast, the docking experiment indicated that **40** did not adapt the desired binding conformation, which could explain its loss of binding affinity.

The correlation in the hinge region was also ambiguous. Our data (not shown) and a previous study [10] demonstrated that the α-methyl of the hinge tryptophan is critical for PD 176,252’s high affinity binding. However, the docking scores of PD 176,252 and its analog without the α-methyl were identical. On the other hand, the cryo-EM structure revealed a hydrogen bond between the hydrogen on the hinge indole and GRPR, which could explain why **28** had a reduction of binding affinity.

In summary, the docking experiment data and our experimental data did not correlate well. While the cryo-EM structure could explain a portion of our experimental findings, it did not account for others. This highlights the structural complexity of GRPR.

## GRPR/NMBR selectivity

We tested the GRPR/NMBR selectivity of our new GRPR antagonists. PD 176,252 was initially discovered as an NMBR antagonist having a seven-fold greater binding affinity for NMBR compared to GRPR [11]. In our IP-One functional assay, PD 176,252 demonstrated a similar four-fold greater potency for NMBR versus GRPR. We tested selected compounds for their antagonist functional potencies against both GRPR and NMBR to determine their GRPR/NMBR selectivity (Table 9). Generally, our compounds also display NMBR selectivity. The only exception was **45**, which displays equal potency for both GRPR and NMBR. Antagonist **45** demonstrated the greatest potency in the functional assays, and it had the best GRPR/NMBR selectivity profile; therefore, we selected it for further investigation.

PD 176,252 was initially discovered as an NMBR antagonist; consequently, our primary off-target evaluation objective was to characterize the NMBR/GRPP selectivity of our antagonists. Once an advanced lead with an optimized in vitro ADME profile is identified, it will be profiled against a panel of GPCRs to evaluate its broader selectivity and off-target activity.

**Table 9:** GRPR/NMBR antagonistic functional selectivity in IP-One assay

1	NMBR/GRPR selectivity*
	0.25x
**6**	0.78x
**7**	0.13x
**12**	0.19x
**40**	0.07x
**45**	1.16x
**46**	0.33x
**53**	0.68x

*By IP-one functional assay

### In vitro ADME Profiles of 45

We compared selected in vitro ADME properties of **45** to those of PD 176,252 (Table 2). Compound **45**’s liver microsomal t_1/2_ was comparable to that of PD 176,252. It also demonstrated similar aqueous solubility, Cl_int_, plasma protein binding, hERG affinity, and CYP inhibition profiles as PD 176,252. These data suggested that the ADME properties were likely rooted in the flexible backbones and high cLogP shared by those two compounds. We need to note that, despite having low aqueous solubility similar to PD 176,252, antagonist **45** did not require extensive formulation like PD 176,252 for in vivo pharmacology studies.

## Pharmacology of 45

**Fig. 2:**
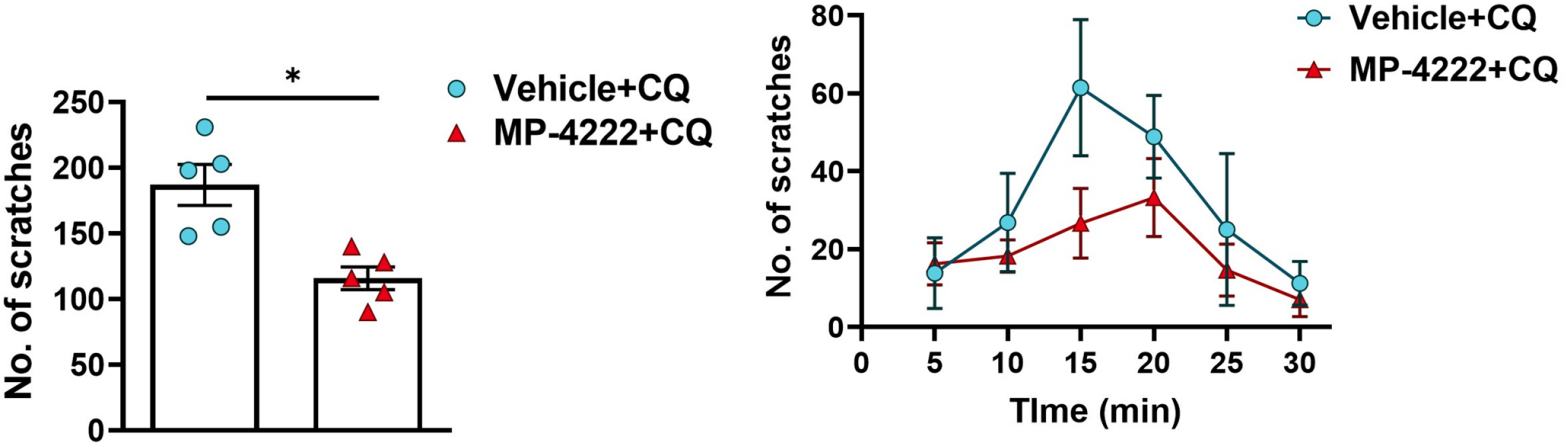
The effect of i.p. injections of **45 (MP-4222)** on CQ-induced itch. Vehicle (5% DMSO) or **45** (10 mg/kg) was pre-injected (40 min) intraperitonially. Scratching behaviors were recorded for 30 min. *n* = 5 mice per group. *p < 0.05, unpaired t test. Data are presented as mean ± SEM

In our previous studies, we demonstrated that chloroquine (CQ) induces histamine-independent itch, and that GRPR knockout mice were insensitive to CQ-induced itch [5]. Compound **45** was evaluated for anti-itching effects in the CQ-induced itch mouse model. At 10 mg/kg IP administration, **45** significantly reduced CQ-induced scratching behavior compared to the vehicle, returning the number of scratches to baseline (without CQ stimulation), which is the theoretical maximum effect of a GRPR antagonist (Fig. 2). The in vivo anti-itch effect demonstrated by **45** was like that of PD 176,252 in our previous experiments (data not shown).

It is noteworthy that both PD 176,252 and **45** required pre-injection to elicit pharmacological effects, and the underlying rationale remains to be elucidated. Furthermore, given the high hepatic metabolism rates for both compounds, neither was likely to sustain therapeutic plasma concentrations beyond 40 min. While investigating the mechanisms is beyond the scope of the present study, further efforts involving radio-labeling an advanced lead to evaluate tissue distribution and related properties are planned. This will facilitate studying the correlation of compound localization with peripheral tissues expressing GRPP.

### Chemistry

**Scheme 1:**
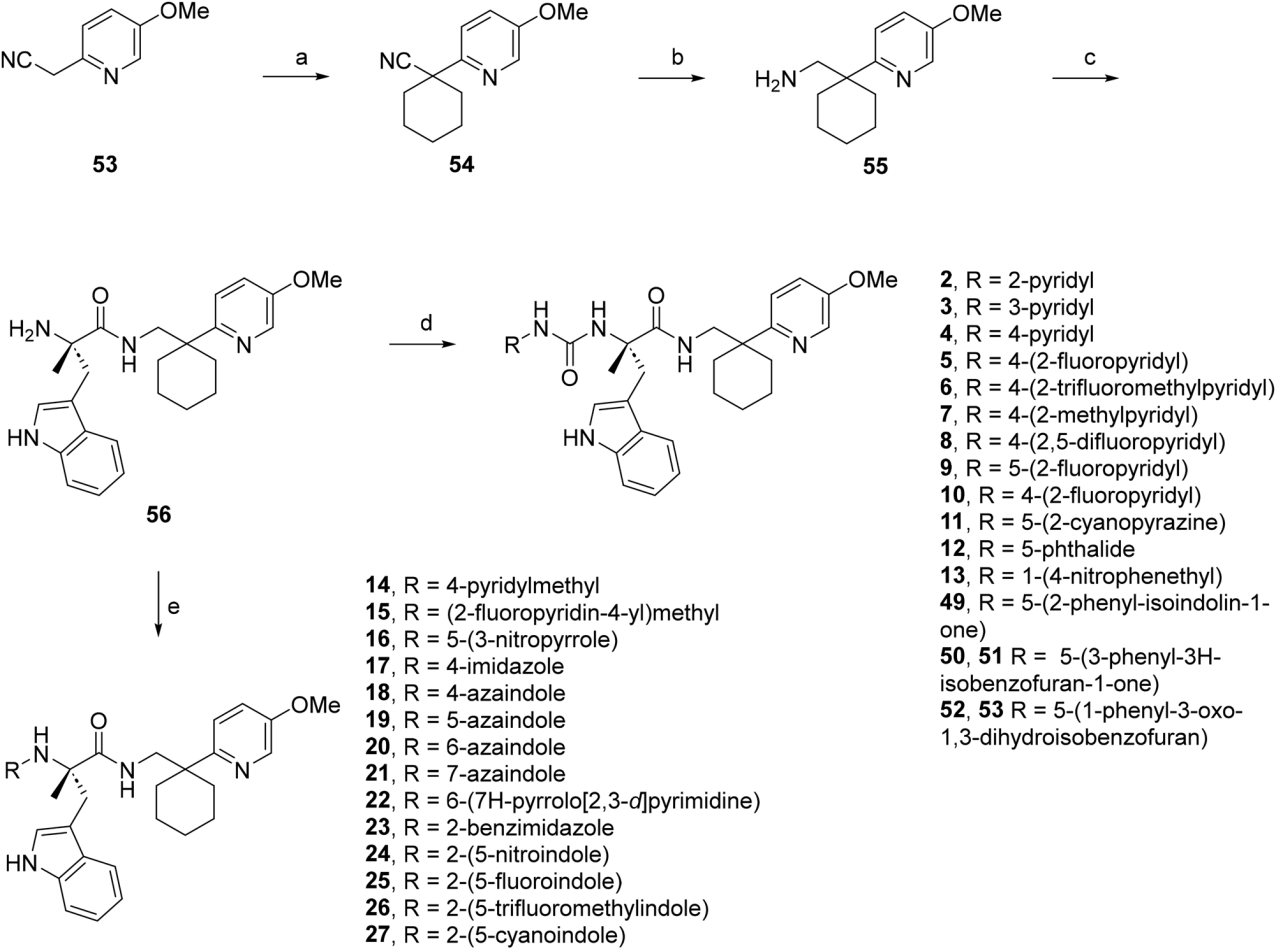
The synthesis of **2**–**13**, **49**–**52** and compound **14**–**27**. Reagents and conditions: (a) 1,5-diiodopentane, NaH, DMSO, THF, 20 °C, 12 h; (b) BH_3_-THF, THF, 25–60 °C, 0.5 h; (c) (S)-2-amino-3-(1 H-indol-3-yl)-2-methylpropanoic acid, HATU, DIEA, DMF, 0 °C, 2 h; (d) isocyanate, TEA, DCM, 20 °C, 1 h; (e) HATU, DIEA, DMF, 0 °C, 2 h

The synthesis of **2**–**13**, **49**–**52** and **14**–**27** is shown in Scheme 1. First, 2-(5-methoxypyridin-2-yl)acetonitrile (**53**) was deprotonated with excess NaH, followed by the addition of one equivalent of 1,5-dibromopentane to form nitrile **54**. This nitrile was then reduced using Raney nickel to obtain amine **55**, which was subsequently coupled with α-methyltryptophan to produce the key intermediate **56**. The various isocyanates that were used were obtained by treating the corresponding commercially available amines with triphosgene; these isocyanates were then reacted with intermediate **56** to form **2**–**13** and **49**–**53.** Additionally, intermediate **56** was coupled with the corresponding commercially available heterocylic carboxylic acids to produce **14**–**27**.

**Scheme 2:**
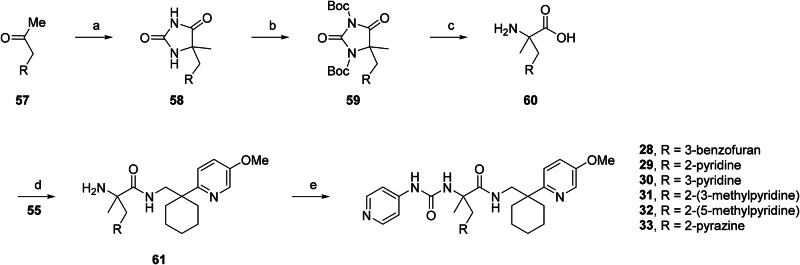
The synthesis of **28**–**33**. Reagents and conditions: (a) (NH_4_)_2_CO_3_, NaCN, MeOH, H_2_O; 100 °C, 17 h (b) (BOC)_2_O, DMAP, THF, 20 °C, 4 h; (c) KOH, THF, H_2_O, 70 °C, 24 h, then HCl, EtOH, 20 °C, 1 h; (d) HATU, DIEA, DMF, 0 °C, 2 h; (e) isocyanate, TEA, DCM, 20 °C, 1 h

Compounds **28**–**33** were synthesized as shown in Scheme 2. First, various racemic α-methyl-amino acids were synthesized using an established method starting from methyl ketones [18]. Briefly, hydantoins **58** were generated from the corresponding methyl ketones, then di-Boc protected, and subsequently hydrolyzed using KOH in THF to yield racemic α-methyl-amino acids **60**. These amino acids **60** were coupled with intermediate **55** to form amines **61**, which were subsequently condensed with 4-isocyanatopyridine, formed by reacting 4-aminopyridine with triphosgene, to produce **28**–**33**.

**Scheme 3:**
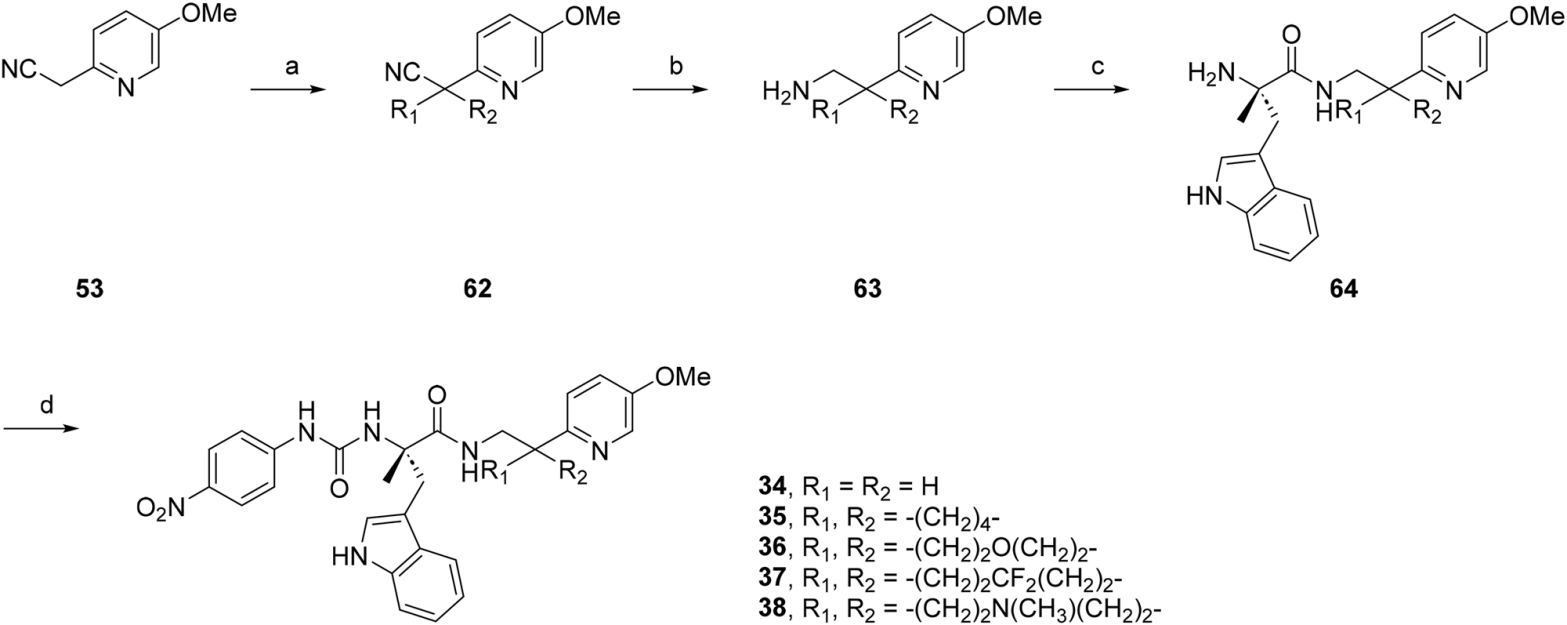
The synthesis of **34**–**38**. Reagents and conditions: (a) dihalides, NaH, DMSO, THF, 20 °C, 12 h; (b) BH_3_-THF, THF, 25–60 °C, 0.5 h; (c) (S)-2-amino-3-(1 H-indol-3-yl)-2-methylpropanoic acid, HATU, DIEA, DMF, 0 °C, 2 h; (d) isocyanate, TEA, DCM, 20 °C, 1 h

Compounds **34**–**38** were synthesized as shown in Scheme 3. 2-(5-Methoxypyridin-2-yl)acetonitrile (**53**) was deprotonated with excess NaH, followed by the addition of one equivalent of dihalides to form nitriles **62**. These nitriles **62** were reduced using Raney nickel to obtain amines **63**, which were subsequently coupled with α-methyltryptophan to produce amines **64**. Amines **64** were then reacted with 2-isocyanato-5-methoxypyridine, formed by reacting 4-nitroaniline with triphosgene, to produce **35**–**38**. For the synthesis of **34**, nitrile **53** was directly reduced using Raney nickel, and the rest of the synthesis followed the same procedure.

**Scheme 4:**
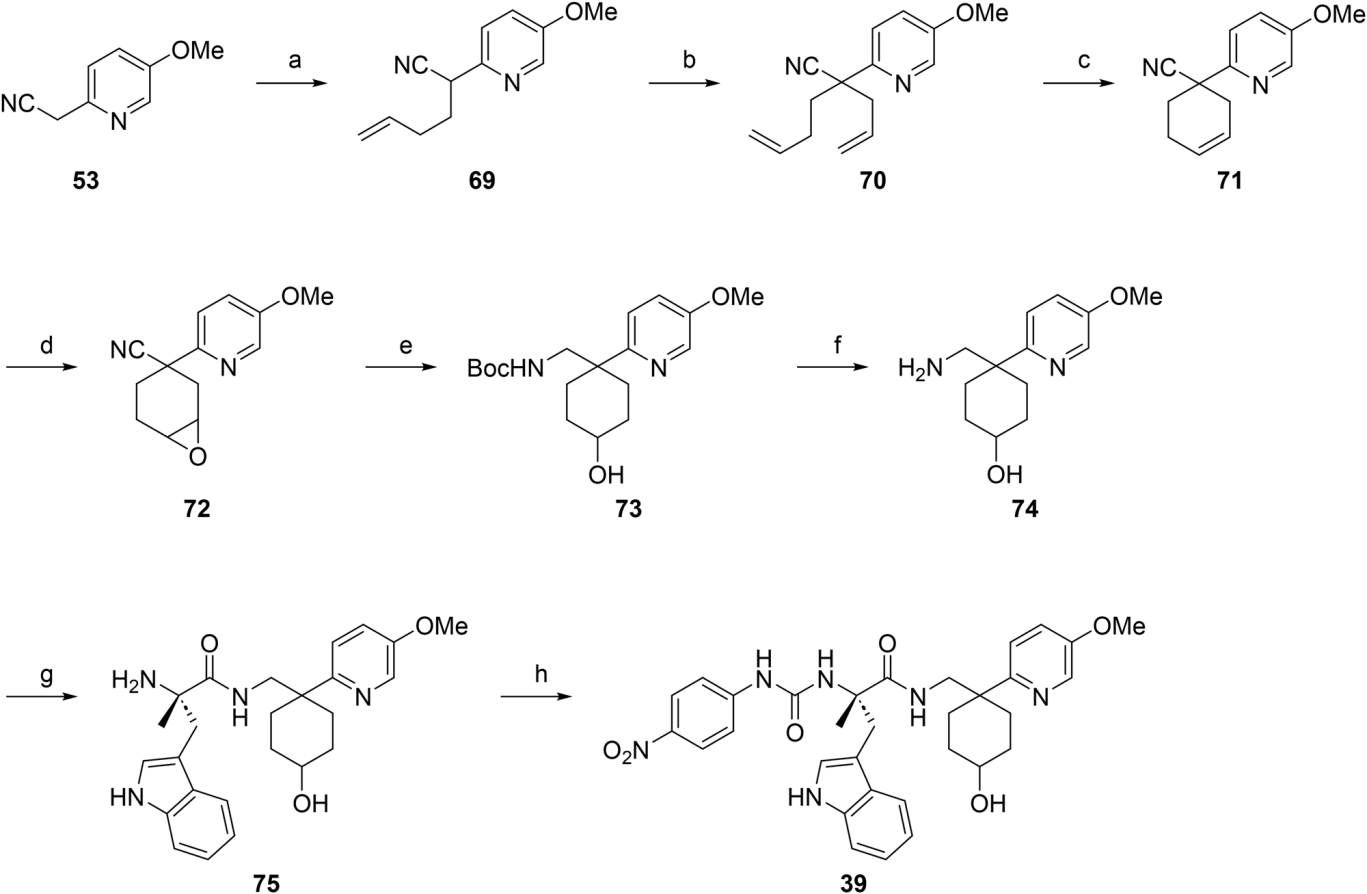
The synthesis of **39**. Reagents and conditions: (a) 4-bromobut-1-ene, NaH, THF, 20–35 °C, 12 h; (b) 3-iodoprop-1-ene, NaH, DMF, 0–20 °C, 12 h; (c) Grubbs 1, Tol., 80 °C, 12 h; (d) m-CPBA, DCM, 0–20 °C, 12 h; (e) H_2_, Pd/C, BOC_2_O, MeOH, 30 °C, 48 h; (f) HCl/EtOAc, 20 °C, 1 h; (g) PyBop, 4-methylmorpholine, THF, 20 °C, 12 h; (h) isocyanate, CH_2_Cl_2_, TEA, 25 °C, 1 h

Compound **39** was synthesized as shown in Scheme 4. 2-(5-Methoxypyridin-2-yl)acetonitrile (**53**) was deprotonated with excess NaH to react with 4-bromo-1-butene to form **69**, which was subsequently deprotonated by excess NaH to react with 3-iodoprop-1-ene to produce diene **70**. Diene **70** underwent intramolecular cyclization using the first-generation Grubbs catalyst to yield cyclohexene **71.** At this stage, the two enantiomers could be separated by chromatography using supercritical fluid (SFC); however, they were used in the following steps without separation. Cyclohexene **71** was oxidized by mCPBA to form epoxide **72**, which was subsequently opened under H_2_ and Pd/C to afford the cyclohexanol **73**. Interestingly, among the six possible cyclohexanol derivatives (from racemic **71**) which could be formed, only one isomer was produced. 2D NMR showed that this isomer has the hydroxy group at the para-position of the disubstituted cyclohexyl carbon. We did not identify the absolute chirality of this carbon. The BOC group on **73** was removed, and the resulting amine **74** followed the same synthetic steps from **64** to **34** to ultimately afford **39**.

**Scheme 5:**
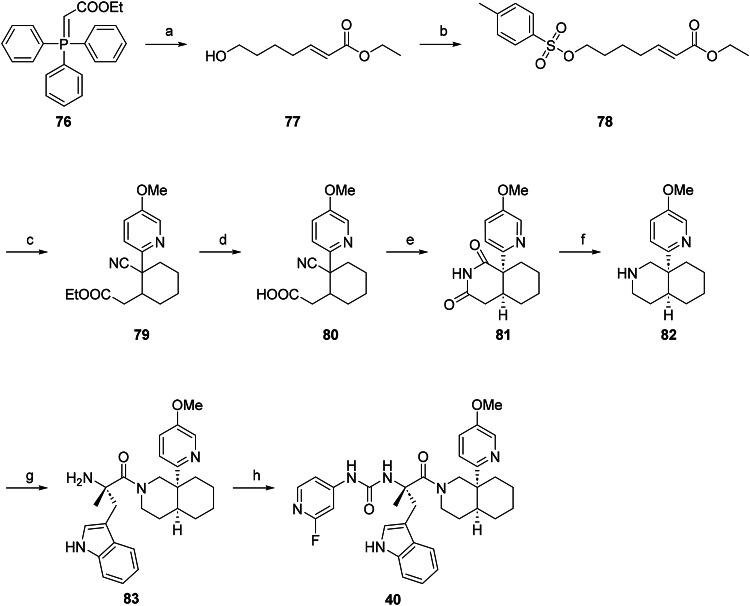
The synthesis of **40**. Reagents and conditions: (a) tetrahydro-2 H-pyran-2-ol, 18-crown-6 ether, DCM, 40 °C,48 h, 79%; (b) TosCl, DMAP, TEA, 20 °C, 24 h, 73%; (c) **53**, NaH, THF, 25–80 °C, 48 h, 82%; (d) NaOH, H_2_O, EtOH, 20 °C, 2 h, 77%; (e) HCl, H_2_O, 100 °C, 12 h, 83%; (f) LiAlH_4_, THF, 80 °C, 12 h, 64%; (g) (S)-2-amino-3-(1 H-indol-3-yl)-2-methylpropanoic acid, HATU, DIEA, DMF, 0–20 °C, 0.5 h, 42%; (h) 2-fluoro-4-isocyanato-pyridine, CH_2_Cl_2_, TEA, 0–20 °C, 0.5 h, 52%

Compound **40** was synthesized as shown in Scheme 5. Ylid **76** underwent Wittig reaction to yield alcohol **77**. Alcohol **77** was then tosylated to yield **78**. This tosylate was subsequently reacted with deprotonated 2-(5-methoxypyridin-2-yl)acetonitrile (**53**) to form ester **79**, which was hydrolyzed to yield carboxylic acid **80**. Under acidic conditions, **80** underwent intramolecular cyclization to form glutarimide **81**, which was then reduced by LAH to yield amine **82**. Amine **82** followed the same synthetic steps from **56** to **5** to ultimately afford **40**.

**Scheme 6:**
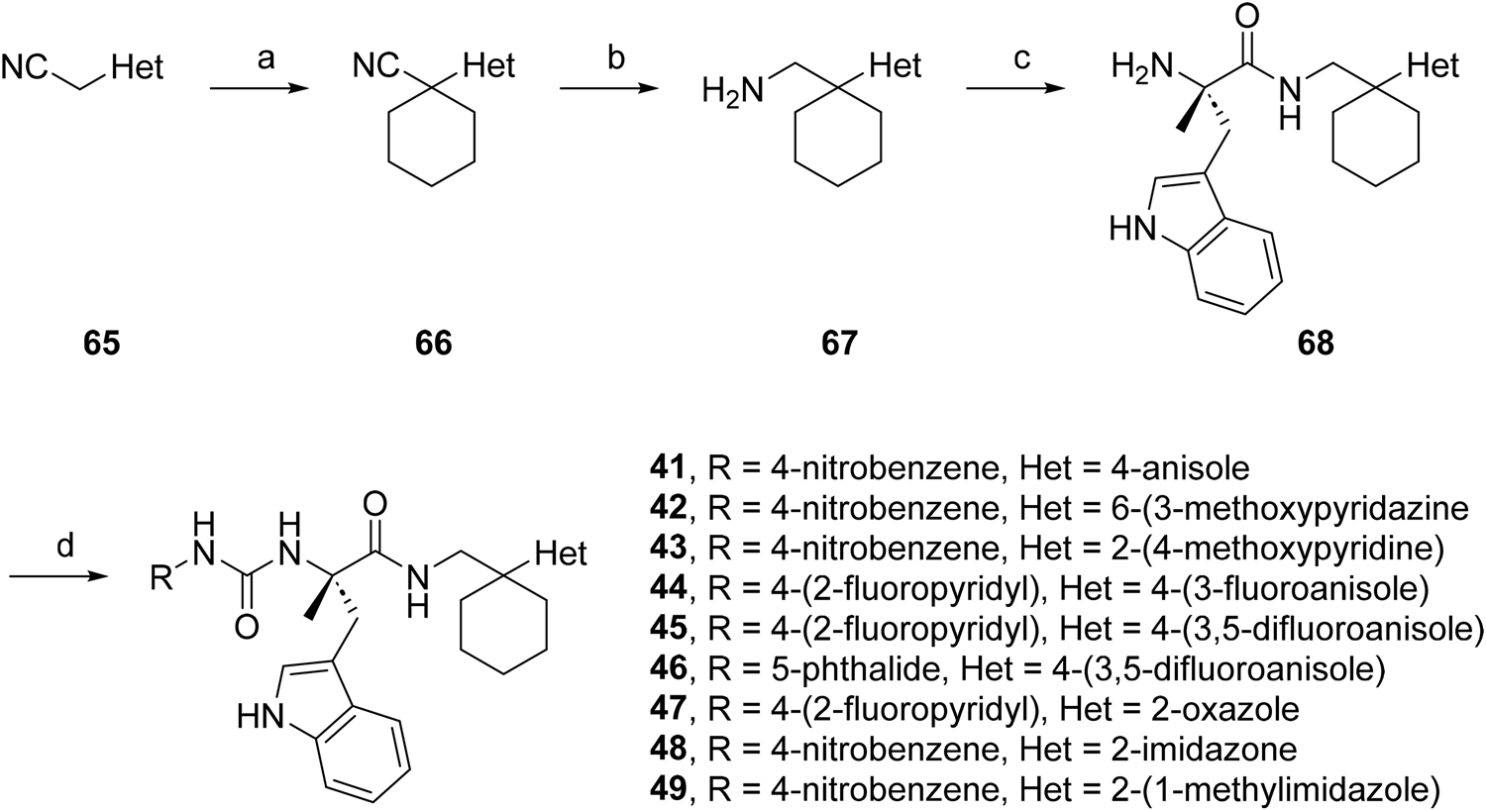
The synthesis of **41**–**49**. Reagents and conditions: (a) 1,5-diiodopentane, NaH, DMSO, THF, 20 °C, 12 h; (b) BH_3_-THF, THF, 25–60 °C, 0.5 h; (c) (S)-2-amino-3-(1 H-indol-3-yl)-2-methylpropanoic acid, HATU, DIEA, DMF, 0 °C, 2 h; (d) isocyanate, TEA, DCM, 20 °C, 1 h

Compounds **41**–**49** were synthesized as shown in Scheme 6. Various commercially available 2-heterocylic-substituted acetonitrile compounds were deprotonated with excess NaH, followed by the addition of one equivalent of 1,5-dibromopentane to form nitrile **66**. Nitrile **66** was then reduced using Raney nickel to obtain amine **67**, which were subsequently coupled with α-methyltryptophan to produce amine **68**. Next, various isocyanates were first formed by reacting the corresponding commercially available amines with triphosgene; these resulting isocyanates were then condensed with amines **68** to afford **41**–**49.**

**Scheme 7:**
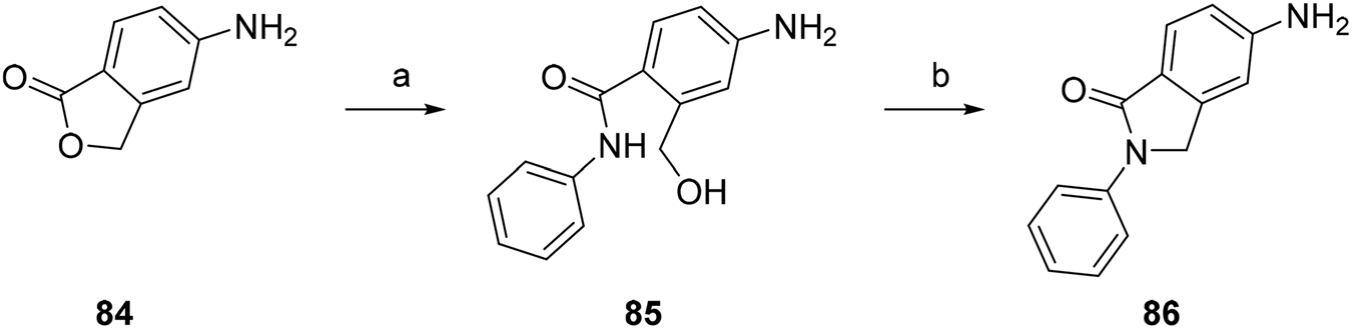
The synthesis of **86**. Reagents and conditions: (a) aniline, AlMe_3_, THF, 0–70 °C, 12 h; (b) PPh_3_, DEAD, THF, 0–20 °C, 12 h

**Scheme 8:**
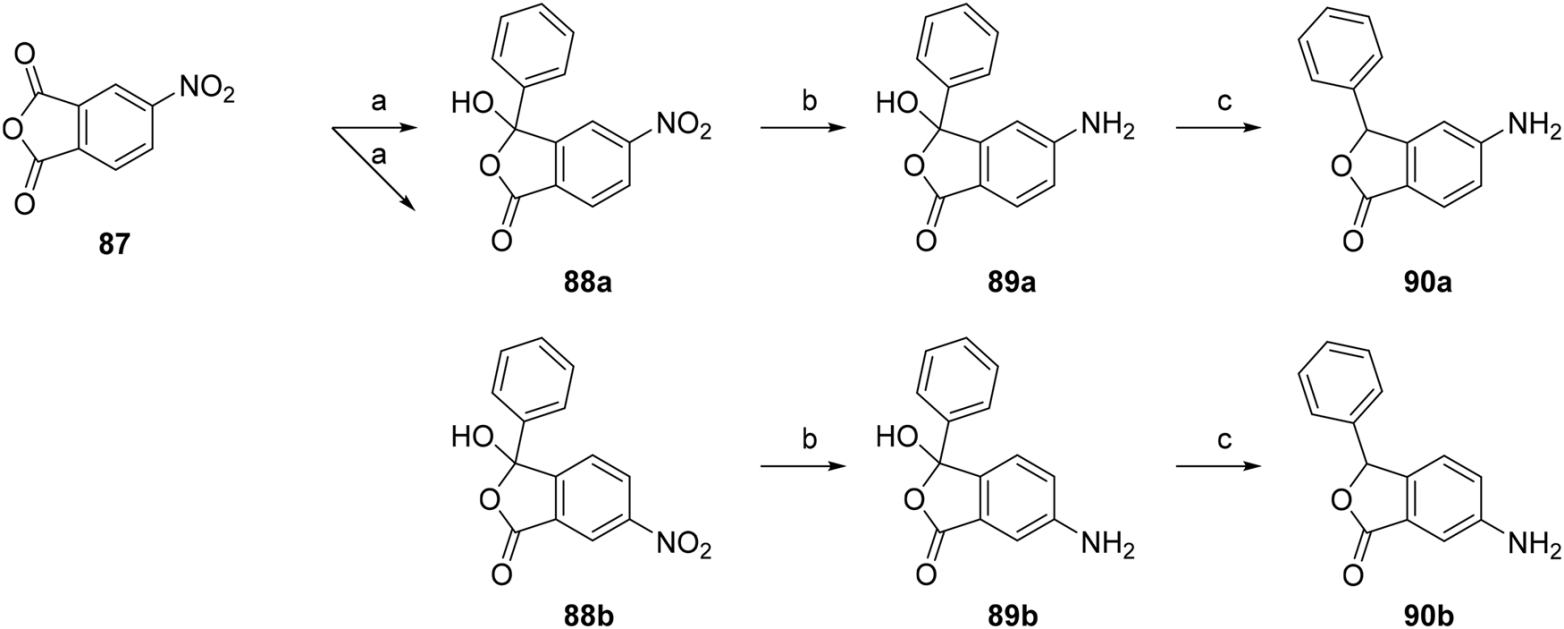
The synthesis of **95a** and **95b**. Reagents and conditions: (a) PhMgBr, THF, 0–20 °C, 1 h; (b) Zn, AcOH, THF, 20 °C, 5 h; (c) Et_3_SiH, TFA, DCM, BF_3_-Et_2_O, 0–20 °C, 12 h

The amines required to synthesize the isocyanates for **50**–**53** were not commercially available and their syntheses are shown in Schemes 7 and 8. 5-Aminophthalide (**84**) was treated with aniline to form **85**. The hydroxyl group of **85** was then activated using PPh_3_ and DEAD at 0 °C and the reaction temperature raised to 20 °C to facilitate intramolecular cyclization to form amine **86**. Additionally, 5-nitroisobenzofuran-1,3-dione (**87**) was treated with Grignard reagent phenylmagnesium bromide to form 2 pairs of enantiomers: **88a** and **88b**. Compounds **88a** and **89b** were separated by HPLC. The nitro groups of **88a** and **88b** were then reduced by Zn/AcOH to produce **89a** and **89b**. Finally, the hydroxyl groups of **89a** and **89b** were removed using triethylsilane and trifluoroacetic acid to afford amines **91a** and **91b**.

## Conclusion

We described the SAR studies for a series of small-molecule GRPR antagonists using PD 176,252 as an initial lead. Our research led to the discovery of **45**, a potent small-molecule GRPR antagonist reported to date. Compared to PD 176,252, **45** was four-fold more potent in the radiobinding assay and equally potent in the IP-One functional assay. And it showed equal selectivity for GRPR/NMBR. Compound **45** demonstrated anti-itch efficacy in our CQ-induced acute itch model. Additionally, we found that the SAR observations could not be fully explained by the published cryo-EM structure of GRPR-PD 176,252, highlighting the structural complexity of GRPR. Lastly, we discovered **53** and **54**, which may have potential to be used for radio-labeling for GRPR imaging applications.

### Experimental

#### Radiobinding assay

Homogenates of HEK293 cells stably transfected with hGRPR (selected with G418) were aliquoted to each well of 96-well plates (~ 52 µg protein per well). The homogenates were incubated with testing compounds or GRP/ PD 176,252 (as positive controls for normalization) and 0.1 nM of radioligand [^125^I]-BN (American Radiolabeled Chemicals, Inc., St. Louis, MO, USA) in a total assay volume of 150 µL at 24 °C in incubation plates (Fisher Scientific, Pittsburgh, PA, USA) for 30 min, in a binding buffer contains 50 mM Tris-HCl, 50 mM NaCl, 33mM EDTA, pH 7.8. At the end of incubation, the contents of each well were transferred to and vacuumed through MultiScreen HTS 96-Well Filter Plates (Sigma-Aldrich) to remove the free radioligands. The filter plates were then washed twice with ice-cold wash buffer containing 10 mM Tris-HCl, 150 mM NaCl, pH 7.4. The fiberglass filters containing homogenates were transferred to MicroBeta counting vials and dissolved in 2 mL biodegradable scintillation cocktail (Research Products International, Mount Prospect, IL, USA). Radioactivity was quantified using a liquid scintillation counter (Hitachi Aloka, LSC8000). Non-specific accumulation of ^125^I-BN was measured in the presence of 10 µM GRP.

Initial screening at two doses, 100 nM and 1 µM, of test compounds was performed to estimate the IC_50_ values. Compounds with IC_50_ < 100 nM were assessed with a full dose competitive binding assay (0.1 nM, 1 nM, 3 nM, 10 nM, 30 nM, 100 nM, 1 µM, 10 µM) and the KaleidaGraph (Synergy Software, Reading, PA, USA) was used for nonlinear regression to derive the accurate IC50 value for each compound as we reported.

#### GRPR and NMBR IP-One Functional Assays (Antagonist Mode)

IP-One antagonist assays were performed according to manufacturer’s instructions (Cisbio). HEK293 cells stably expressing GRPR were trypsinized and resuspended in Stimulation buffer and seeded into 384-well Proxiplate Plus plates pre-dispensed with an eight-point four-fold serial dilution of test compounds, tested in quadruplicate. Cells were incubated with the compounds for 30 min at 37 °C after which, GRP was added and followed by incubation at 37 °C for 60 min. IP-One detection reagents were then dispensed into the wells and incubated for 1.5 h at RT. Fluorescence measurements (Ex320/Em620 and Ex320/Em665) were performed with the Envision plate reader. HTRF ratio data (Em665/Em620) were converted into IP1 concentrations using an IP1 standard curve and then normalized to plate controls to determine % inhibition. Wells containing DMSO alone and treated with or without GRP served as the negative and positive controls (representing 0% and 100% inhibition, respectively). % inhibition data were plotted in GraphPad Prism and the IC_50_s were determined using non-linear regression analysis.

Similar procedures were performed for the NMBR functional assays, with two key changes: Neuromedin B was used as the agonist. For the cells, division-arrested cells (Multispan) were used directly upon thawing and resuspension in Stimulation buffer.

#### Molecular modeling

The computational docking experiment was conducted using the Molecular Operating Environment (MOE) 2024.0601 Chemical Computing Group ULC, 910–1010 Sherbrooke St. W., Montreal, QC H3A 2R7, 2025.

#### Synthesis

All reagents were of commercial grade and were used as received without further purification, unless otherwise stated. Commercially available anhydrous solvents were used for reactions conducted under inert atmosphere. Reagent grade solvents were used in all other cases, unless otherwise specified. Yields were not optimized.

##### *(S)-2-Amino-3-(1 H-indol-3-yl)-N-((1-(5-methoxypyridin-2-yl)cyclohexyl)methyl)-2-methylpropanamide* (**56**).

To a solution of (S)-2-amino-3-(1 H-indol-3-yl)-2-methylpropanoic acid (150 mg, 687.28 µmol, 1 *eq*) and (1-(5-methoxypyridin-2-yl)cyclohexyl)methanamine **55** (166.56 mg, 756.01 µmol, 1.1 *eq*) in DMSO (2 mL) was added DIEA (133.24 mg, 1.03 mmol, 179.57 µL, 1.5 *eq*) and HATU (391.99 mg, 1.03 mmol, 1.5 *eq*) at 20 °C. The mixture was stirred at 20 °C for 2 h. The reaction mixture was filtered and concentrated under reduced pressure to give a residue. The residue was purified by prep-HPLC (basic condition, column: Waters Xbridge Prep OBD C18 150*40 mm*10um; mobile phase: [water (0.05% NH_3_H_2_O+10mM NH_4_HCO_3_)-ACN]; B%: 25%-55%, 8 min) to give **56** (190 mg, 451.79 µmol, 65.74% yield) as a light yellow solid. MS: *m/z* 421.2 (M + H)^+^.

##### *(2 S)-3-(1 H-indol-3-yl)-N-((1-(5-methoxypyridin-2-yl)cyclohexyl)methyl)-2-methyl-2-(3-(1-oxo-3-phenyl-1*,*3-dihydroisobenzofuran-5-yl)ureido)propanamide* (**12**).

To a solution of (S)-2-amino-3-(1H-indol-3-yl)-N-((1-(5-methoxypyridin-2-yl)cyclohexyl)methyl)-2-methylpropanamide (**56**, 50 mg, 118.89 µmol, 1 *eq*) and 5-isocyanatoisobenzofuran-1(3 H)-one (52.06 mg, 297.23 µmol, 2.5 *eq*) in THF (2 mL) was added [dibutyl(dodecanoyloxy)stannyl] dodecanoate (75.09 mg, 118.89 µmol, 1 *eq*). The mixture was stirred at 15 °C for 0.5 h. The mixture was added water and concentrated in vacuum to give a residue. The residue was purified by prep-HPLC (FA condition: column: 3_Phenomenex Luna C18 75*30 mm*3um; mobile phase: [water (0.2% FA)-ACN]; B%: 10%-45%, 10 min) to give **12** (20 mg, 33.57 µmol, 28.24% yield, 100% purity) as a white solid. MS: *m/z* 596.3 (M + H)^+^. ^1^H NMR (400 MHz, METHANOL-d4) *δ* ppm 3.43 (br d, 1H, *J* = 8.8 Hz), 3.31 (br s, 1H), 3.18 (s, 3 H), 2.27 (br d, 1H, *J* = 11.6 Hz), 2.15–2.09 (m, 1H), 1.86–1.68 (m, 4 H), 1.40–1.30 (m, 2 H).

##### *(S)-2-Amino-N-(((1s*,*4R)-4-hydroxy-1-(5-methoxypyridin-2-yl)cyclohexyl)methyl)-3-(1 H-indol-3-yl)-2-methylpropanamide* (**75**).

To a solution of (1s,4s)-4-(aminomethyl)-4-(5-methoxypyridin-2-yl)cyclohexanol (**74**, 38.98 mg, 164.97 µmol, 1.1 *eq*, HCl) in THF (2 mL) was added (S)-2-amino-3-(1 H-indol-3-yl)-2-methylpropanoic acid (32.73 mg, 149.98 µmol, 1 *eq*), PYBOP (117.07 mg, 224.96 µmol, 1.5 *eq*) and 4-methylmorpholine (60.68 mg, 599.91 µmol, 65.96 µL, 4 *eq*). The mixture was stirred at 25 °C for 12 h. The reaction mixture was concentrated under reduced pressure to remove solvent. The residue was purified by prep-TLC (SiO_2_, DCM: MeOH = 10:1) to give **75** (50 mg, 114.54 µmol, 76.37% yield) as a yellow gum. MS: *m/z* 437.3 (M + H)^+^.

##### *(S)-2-(3-(2-Fluoropyridin-4-yl)ureido)-N-(((1s*,*4R)-4-hydroxy-1-(5-methoxypyridin-2-yl)cyclohexyl)methyl)-3-(1 H-indol-3-yl)-2-methylpropanamide* (**39**).

To a solution of (S)-2-amino-N-(((1s,4R)-4-hydroxy-1-(5-methoxypyridin-2-yl)cyclohexyl)methyl)-3-(1H-indol-3-yl)-2-methylpropanamide (**75**, 40.00 mg, 91.63 µmol, 1 *eq*) in DCM (2 mL) was added TEA (27.82 mg, 274.89 µmol, 38.26 µL, 3 *eq*) and 2-fluoro-4-isocyanatopyridine (25.31 mg, 183.26 µmol, 2 *eq*). The mixture was stirred at 25 °C for 1 hr. The reaction mixture was concentrated under reduced pressure to remove solvent. The residue was purified by prep-HPLC (column: Phenomenex Gemini-NX C18 75*30 mm*3um; mobile phase: [water (10 mM NH_4_HCO_3_)-ACN]; B%: 10%-40%,12 min) to give **39** (5 mg, 8.70 µmol, 9.50% yield, 100% purity) as a white solid. MS: *m/z* 596.3 (M + H)^+^. ^1^H NMR (400 MHz, METHANOL-d4) *δ* ppm 8.06 (d, 1H, *J =* 2.8 Hz), 7.97 (d, 1H, *J =* 5.6 Hz), 7.47 (d, 1H, *J =* 8.0 Hz), 7.35–7.30 (m, 2 H), 7.25 (d, 1H, *J =* 1.6 Hz), 7.15–7.10 (m, 2 H), 7.08–7.03 (m, 2 H), 6.92 (t, 1H, *J =* 7.6 Hz), 3.70 (s, 3 H), 3.64–3.54 (m, 1H), 3.48 (d, 1H, *J =* 14.8 Hz), 3.25–3.12 (m, 3 H), 2.45–2.26 (m, 2 H), 1.75 (br s, 2 H), 1.52 (s, 3 H), 1.50–1.41 (m, 1H), 1.34–1.27 (m, 1H), 1.10 (br d, 2 H, *J =* 4.0 Hz).

##### *(E)-Ethyl 7-hydroxyhept-2-enoate* (**77**).

To a solution of ethyl 2-(triphenylphosphoranylidene)acetate (**76**, 4.78 g, 13.71 mmol, 1.4 *eq*) and tetrahydropyran-2-ol (1 g, 9.79 mmol, 1 *eq*) in DCM (150 mL) was added 1,4,7,10,13,16-hexaoxacyclooctadecane (258.80 mg, 979.13 µmol, 0.1 *eq*) and K_2_CO_3_ (2.71 g, 19.58 mmol, 2 *eq*). The mixture was stirred at 40 °C for 48 h. The reaction mixture was filtered and concentrated under reduced pressure to give a residue. The residue was purified by column chromatography (SiO_2_, Petroleum ether/Ethyl acetate = 4/1 to 1/1) to give **77** (1.34 g, 7.78 mmol, 79.47% yield) as a pale yellow solid.

##### *(E)-Ethyl 7-(tosyloxy)hept-2-enoate* (**78**).

To a solution of (*E*)-ethyl 7-hydroxyhept-2-enoate (**77**, 1.3 g, 7.55 mmol, 1 *eq*) and 4-methylbenzenesulfonyl chloride (1.73 g, 9.06 mmol, 1.2 *eq*) in DCM (25 mL) was added TEA (1.53 g, 15.10 mmol, 2.10 mL, 2 *eq*) and DMAP (92.22 mg, 754.84 µmol, 0.1 *eq*). The mixture was stirred at 20 °C for 24 h. The reaction mixture was concentrated under reduced pressure to remove solvent. The residue was purified by prep-TLC (SiO_2_, Petroleum ether/Ethyl acetate = 5/1 to 1/1) to give **78** (1.8 g, 5.51 mmol, 73.06% yield) as a pale yellow oil. ^1^H NMR (400 MHz, CHLOROFORM-d) *δ* ppm 7.72 (d, 2 H, *J =* 8.4 Hz), 7.28 (d, 2 H, *J =* 8.0 Hz), 6.79 (td, 1H, *J =* 7.2, 15.6 Hz), 5.70 (d, 1H, *J =* 15.6 Hz), 4.11 (q, 2 H, *J =* 7.2 Hz), 3.96 (t, 2 H, *J =* 6.4 Hz), 2.39 (s, 3 H), 2.09 (q, 2 H, *J =* 7.2 Hz), 1.65–1.55 (m, 2 H), 1.45–1.39 (m, 2 H), 1.25–1.16 (m, 4 H).

##### *2-(2-Cyano-2-(5-methoxypyridin-2-yl)cyclohexyl)acetate* (**79**).

To a mixture of NaH (97.18 mg, 2.43 mmol, 60% purity, 1.2 *eq*) in THF (25 mL) was added 2-(5-methoxypyridin-2-yl)acetonitrile (**53**, 300 mg, 2.02 mmol, 1 *eq*) under N_2_, and then the mixture was stirred at 20 °C for 1 h under N_2_. And then was added (*E*)-ethyl 7-(tosyloxy)hept-2-enoate (**78**, 727.01 mg, 2.23 mmol, 1.1 *eq*) under N_2_, the mixture was stirred at 80 °C for 47 h under N_2_ atmosphere. The reaction mixture was quenched by addition NH_4_Cl sat.aq. 50 mL at 0 °C, and then extracted with EtOAc 120 mL (40 mL * 3), dried over Na_2_SO_4_, filtered and concentrated under reduced pressure to give a residue. The residue was purified by prep-TLC (SiO_2_, Petroleum ether/Ethyl acetate = 5/1 to 1/1) to give **79** (500 mg, 1.65 mmol, 81.67% yield) as a pale yellow oil. MS: *m/z* 303.4 (M + H)^+^.

##### *2-(2-Cyano-2-(5-methoxypyridin-2-yl)cyclohexyl)acetic acid* (**80**).

To a solution of ethyl 2-(2-cyano-2-(5-methoxypyridin-2-yl)cyclohexyl)acetate (**79**, 460 mg, 1.52 mmol, 1 *eq*) in EtOH (4 mL) was added NaOH (4 M, 2.5 mL, 6.57 *eq*). The mixture was stirred at 20 °C for 2 h. The reaction mixture was quenched by addition HCl (1 M) 15 mL at 0 °C (PH = 2), and then concentrated under reduced pressure to remove solvent. The residue was diluted with H_2_O 10 mL and extracted with EtOAc 45 mL (15 mL * 3), dried over Na_2_SO_4_, filtered and concentrated under reduced pressure to give **80** (320 mg, 1.17 mmol, 76.68% yield) as a yellow gum. MS: *m/z* 275.0 (M + H)^+^. ^1^H NMR (400 MHz, CHLOROFORM-d) *δ* ppm 8.26 (br s, 1H), 7.46 (br d, 1H, *J =* 8.0 Hz), 7.19 (br d, 1H, *J =* 6.8 Hz), 3.85 (s, 3 H), 2.64–2.49 (m, 1H), 2.01–1.92 (m, 2 H), 1.86 (br d, 2 H, *J =* 7.2 Hz), 1.81–1.59 (m, 4 H), 1.29 (br t, 2 H, *J =* 7.2 Hz).

##### *(4aS*,*8aS)-8a-(5-Methoxypyridin-2-yl)hexahydroisoquinoline-1*,*3(2 H*,*4 H)-dione* (**81**).

A mixture of 2-(2-cyano-2-(5-methoxypyridin-2-yl)cyclohexyl)acetic acid (**80**, 300 mg, 1.09 mmol, 1 *eq*) in HCl (30 mL, 37% purity) was stirred at 100 °C for 12 h. The reaction mixture was concentrated under reduced pressure to remove HCl. The residue was diluted with Na_2_CO_3_ sat.aq. 20 mL and extracted with EtOAc 60 mL (20 mL * 3), dried over Na_2_SO_4_, filtered and concentrated under reduced pressure to give a residue. The residue was purified by prep-HPLC (column: Waters Xbridge Prep OBD C18 150*40 mm*10um; mobile phase: [water (0.05% NH_3_H_2_O + 10 mM NH_4_HCO_3_)-ACN]; B%: 15%-40%,8 min) to give **81** (250 mg, 911.36 µmol, 83.33% yield) as a yellow oil. MS: *m/z* 275.0 (M + H)^+^. ^1^H NMR (400 MHz, CHLOROFORM-d) *δ* ppm 8.25 (d, 1H, *J =* 2.8 Hz), 7.94 (br s, 1H), 7.25–7.20 (m, 1H), 7.20–7.14 (m, 1H), 3.86 (s, 3 H), 2.62 (dd, 1H, *J =* 5.2, 18.0 Hz), 2.52 (br d, 1H, *J =* 13.2 Hz), 2.33 (br d, 1H, *J =* 17.6 Hz), 1.89–1.78 (m, 3 H), 1.73 (br d, 1H, *J =* 11.6 Hz), 1.59 (br t, 1H, *J =* 13.2 Hz), 1.47–1.31 (m, 3 H).

##### *(4aS*,*8aR)-8a-(5-Methoxypyridin-2-yl)decahydroisoquinoline* (**82**).

To a mixture of LiAlH_4_ (48.43 mg, 1.28 mmol, 2.5 *eq*) in THF (4 mL) was added (4aS,8aS)-8a-(5-methoxypyridin-2-yl)hexahydroisoquinoline-1,3(2 H,4 H)-dione (**81**, 140 mg, 510.36 µmol, 1 *eq*) at 0 °C under N_2_ atmosphere, and then the mixture was stirred at 80 °C for 2 h under N_2_ atmosphere. The reaction mixture was quenched by addition H_2_O 3 mL at 0 °C, and then was added NaOH (1 N) 3 mL and H_2_O 9 mL, the mixture was filtered under reduced pressure and the filtrate was extracted with EtOAc 30 mL (10 mL * 3), dried over MgSO_4_, filtered and concentrated under reduced pressure to give (4aS,8aR)-8a-(5-methoxypyridin-2-yl)decahydroisoquinoline **82** (80 mg, 324.75 µmol, 63.63% yield) as a yellow gum. MS: *m/z* 247.2 (M + H)^+^.

##### *1-((S)-3-(1 H-indol-3-yl)-1-((4aS*,*8aR)-8a-(5-methoxypyridin-2-yl)octahydroisoquinolin-2(1 H)-yl)-2-methyl-1-oxopropan-2-yl)-3-(2-fluoropyridin-4-yl)urea* (**40**).

To a solution of 2-fluoro-4-isocyanato-pyridine (12.37 mg, 89.57 µmol, 2 *eq*) in DCM (2 mL) was added TEA (11.33 mg, 111.96 µmol, 15.58 µL, 2.5 *eq*) and (*S*)-2-amino-3-(1H-indol-3-yl)-1-((4aS,8aR)-8a-(5-methoxypyridin-2-yl)octahydroisoquinolin-2(1H)-yl)-2-methylpropan-1-one (**83**, 20 mg, 44.78 µmol, 1 *eq*) at 0 °C. The mixture was stirred at 20 °C for 10 min. The reaction mixture was blown dry under N_2_ to remove solvent. The residue was purified by prep-HPLC (column: Phenomenex Gemini-NX C18 75*30 mm*3um; mobile phase: [water (0.05%NH_3_H_2_O+10mM NH_4_HCO_3_)-ACN]; B%: 30%-70%,8 min) to give **40** (14 mg, 23.43 µmol, 52.32% yield, 97.862% purity) as a yellow solid. MS: *m/z* 585.1 (M + H)^+^. ^1^H NMR (400 MHz, METHANOL-d4) *δ* ppm 8.23 (d, 1H, *J =* 2.8 Hz), 7.94 (d, 1H, *J =* 5.6 Hz), 7.53 (br d, 1H, *J =* 8.8 Hz), 7.47 (br d, 1H, *J =* 7.2 Hz), 7.40–7.29 (m, 3 H), 7.11–7.00 (m, 3 H), 6.84 (t, 1H, *J =* 7.6 Hz), 4.19 (br s, 1H), 3.92–3.75 (m, 4 H), 3.21 (br d, 2 H, *J =* 14.4 Hz), 2.77 (br s, 2 H), 2.26–1.80 (m, 3 H), 1.70–1.52 (m, 1H), 1.50–1.17 (m, 10 H).

##### *1-(2-Fluoro-4-methoxyphenyl)cyclohexanecarbonitrile* (**66**[**44**]).

To a mixture of NaH (484.37 mg, 12.11 mmol, 60% purity, 2 *eq*) in DMSO (7 ml), then was added 1,5-diiodopentane (1.96 g, 6.05 mmol, 900.32 µL, 1 *eq*) and 2-(2-fluoro-4-methoxyphenyl)acetonitrile (**65**[**44**], 1 g, 6.05 mmol, 1 *eq*) in THF (10 mL) and DMSO (3 mL), the mixture was stirred at 20 °C for 12 h under N_2_ atmosphere. The reaction mixture was quenched by addition saturated aqueous NH_4_Cl solution (100 mL) at 0 °C, and then extracted with EtOAc (50 mL * 3). The combined organic layers were dried over Na_2_SO_4_, filtered and concentrated under reduced pressure to give a residue. The residue was purified by column chromatography on silica gel (PE: EtOAc = 100:1/20:1) to give **66**[**44**] (575 mg, 2.47 mmol, 40.87% yield) as a white oil.

##### *(1-(2-Fluoro-4-methoxyphenyl)cyclohexyl)methanamine* (**67**[**44**]).

To a mixture of 1-(2-fluoro-4-methoxyphenyl)cyclohexanecarbonitrile (**66**[**44**], 300 mg, 1.29 mmol, 1 *eq*) in THF (5 mL) was added BH_3_.THF (1 M, 5 mL, 3.89 *eq*) at 25 °C, the mixture was stirred at 60 °C for 0.5 h under N_2_ atmosphere. The reaction mixture was quenched with MeOH (7 ml) at 0 °C and the reaction mixture was concentrated under reduced pressure to give **67**[**44**] (280 mg, crude) was obtained as a white oil. MS: *m/z* 238.2 (M + H)^+^.

##### *(S)-2-Amino-N-((1-(2-fluoro-4-methoxyphenyl)cyclohexyl)methyl)-3-(1 H-indol-3-yl)-2-methylpropanamide* (**68**[**44**]).

To a mixture of (S)-2-amino-3-(1 H-indol-3-yl)-2-methylpropanoic acid (214.59 mg, 983.23 µmol, 1 *eq*) in DMF (10 mL) was added DIEA (317.69 mg, 2.46 mmol, 428.15 µL, 2.5 *eq*) and (1-(2-fluoro-4-methoxyphenyl)cyclohexyl)methanamine (**67**[**44**], 280 mg, 1.18 mmol, 1.2 *eq*), then was added HATU (560.78 mg, 1.47 mmol, 1.5 *eq*) at 0 °C, the mixture was stirred at 0 °C for 2 h under N_2_ atmosphere. The reaction mixture was quenched by addition H_2_O (10 mL), and then extracted with EtOAc 30 mL (10 mL * 3). The combined organic layers were dried over Na_2_SO_4_, filtered and concentrated under reduced pressure to give a residue. The residue was purified by prep-HPLC (column: Phenomenex Luna C18 200*40 mm*10um; mobile phase: [water (0.2% FA)- ACN]; B%: 10%-50%, 8 min) to give **68**[**44**] (20 mg, 41.36 µmol, 4.21% yield, FA) as a white solid. MS: *m/z* 438.2 (M + H)^+^.

##### *(S)-N-((1-(2-Fluoro-4-methoxyphenyl)cyclohexyl)methyl)-2-(3-(2-fluoropyridin-4-yl)ureido)-3-(1 H-indol-3-yl)-2-methylpropanamide* (**44**).

To a solution of 2-fluoro-4-isocyanato-pyridine (6.43 mg, 46.53 µmol, 1.5 *eq*) and (S)-2-amino-N-((1-(2-fluoro-4-methoxyphenyl)cyclohexyl)methyl)-3-(1H-indol-3-yl)-2-methylpropanamide (**68**[**44**], 15 mg, 31.02 µmol, 1 *eq*, FA) in DCM (1 mL) was added TEA (7.85 mg, 77.55 µmol, 10.79 µL, 2.5 *eq*). The mixture was stirred at 25 °C for 10 min. The reaction mixture was concentrated under reduced pressure to remove solvent. The residue was purified by prep-HPLC (column: Waters Xbridge BEH C18 100*30 mm*10um; mobile phase: [water (10mM NH_4_HCO_3_)-ACN]; B%: 30%-65%, 8 min) to give **44** (5 mg, 8.67 µmol, 27.95% yield, 99.798% purity) as a yellow gum. MS: *m/z* 576.1 (M + H)^+^. ^1^H NMR (400 MHz, DMSO-d6) *δ* ppm 10.81 (s, 1H), 9.50 (s, 1H), 7.98 (d, 1H, *J =* 6.0 Hz), 7.46–7.37 (m, 2 H), 7.32–7.25 (m, 2 H), 7.13–7.04 (m, 2 H), 6.99 (t, 1H, *J =* 7.6 Hz), 6.93 (d, 1H, *J =* 2.0 Hz), 6.83 (t, 1H, *J =* 7.2 Hz), 6.67 (dd, 1H, *J =* 2.4, 14.8 Hz), 6.55 (s, 1H), 6.46 (dd, 1H, *J =* 2.4, 8.8 Hz), 3.68 (s, 3 H), 3.44–3.41 (m, 1H), 3.38 (br d, 2 H, *J =* 5.2 Hz), 3.12 (br dd, 1H, *J =* 5.2, 13.2 Hz), 2.11 (br d, 2 H, *J =* 6.0 Hz), 1.51 (br s, 4 H), 1.41 (s, 3 H), 1.28–1.14 (m, 4 H).

##### *1-(2*,*6-Difluoro-4-methoxyphenyl)cyclohexanecarbonitrile* (**66**[**45**]).

To a mixture of NaH (218.39 mg, 5.46 mmol, 60% purity, 2 *eq*) in DMSO (7 ml), then was added 2-(2,6-difluoro-4-methoxyphenyl)acetonitrile (**65**[**45**], 500 mg, 2.73 mmol, 1 *eq*) and 1,5-diiodopentane (884.34 mg, 2.73 mmol, 406.22 µL, 1 *eq*) in THF (10 mL) and DMSO (3 mL), and then the mixture was stirred at 20 °C for 12 h under N_2_ atmosphere. The reaction mixture was quenched by addition saturated aqueous NH_4_Cl solution (30 mL) at 0 °C, and then extracted with EtOAc (30 mL * 3). The combined organic layers were dried over Na_2_SO_4_, filtered and concentrated under reduced pressure to give a residue. The residue was purified by column chromatography on silica gel (PE: EtOAc = 100:1/20:1) to give **66**[**45**] (380 mg, 1.51 mmol, 55.40% yield) as a white solid.

##### *(1-(2*,*6-Difluoro-4-methoxyphenyl)cyclohexyl)methanamine* (**67**[**45**]).

To a mixture of 1-(2,6-difluoro-4-methoxyphenyl)cyclohexanecarbonitrile (**66**[**45**], 380 mg, 1.51 mmol, 1 *eq*) in THF (5 mL) was added BH_3_.THF (1 M, 5 mL, 3.31 *eq*) at 25 °C, the mixture was stirred at 60 °C for 0.5 h under N_2_ atmosphere. The reaction mixture was quenched with MeOH (8 mL) at 0 °C and the reaction mixture was concentrated under reduced pressure to give **67**[**45**] (300 mg, crude) was obtained as a white solid. MS: *m/z* 256.2 (M + H)^+^.

##### *(S)-2-Amino-N-((1-(2*,*6-difluoro-4-methoxyphenyl)cyclohexyl)methyl)-3-(1 H-indol-3-yl)-2-methylpropanamide* (**68**[**45**]).

To a mixture of (1-(2,6-difluoro-4-methoxyphenyl)cyclohexyl)methanamine (**67**[**45**], 290 mg, 1.14 mmol, 1.2 *eq*) and (S)-2-amino-3-(1 H-indol-3-yl)-2-methylpropanoic acid (206.59 mg, 946.59 µmol, 1 *eq*) in DMF (5 mL) was added DIEA (305.85 mg, 2.37 mmol, 412.20 µL, 2.5 *eq*), then was added HATU (431.91 mg, 1.14 mmol, 1.2 *eq*) at 0 °C, the mixture was stirred at 25 °C for 1 h. The reaction mixture was quenched by addition H_2_O (5 mL), and then extracted with EtOAc 15 ml (5 mL * 3). The combined organic layers were dried over Na_2_SO_4_, filtered and concentrated under reduced pressure to give a residue. The residue was purified by prep-HPLC (column: Waters Xbridge Prep OBD C18 150*40 mm*10um; mobile phase: [water (0.05% NH_3_H_2_O + 10 mM NH_4_HCO_3_)-ACN]; B%: 45%-65%,8 min) to give **68**[**45**] (80 mg, 175.62 µmol, 18.55% yield) as a white solid. MS: *m/z* 456.2 (M + H)^+^.

##### *(S)-N-((1-(2*,*6-Difluoro-4-methoxyphenyl)cyclohexyl)methyl)-2-(3-(2-fluoropyridin-4-yl)ureido)-3-(1 H-indol-3-yl)-2-methylpropanamide* (**45**).

To a mixture of 2-fluoro-4-isocyanato-pyridine (15.78 mg, 114.27 µmol, 1 *eq*) in DCM (1 mL) was added (S)-2-amino-N-((1-(2,6-difluoro-4-methoxyphenyl)cyclohexyl)methyl)-3-(1H-indol-3-yl)-2-methylpropanamide (**68**[**45**], 52.06 mg, 114.27 µmol, 1 *eq*) and TEA (11.56 mg, 114.27 µmol, 15.91 µL, 1 *eq*) at 0 °C, the mixture was stirred at 25 °C for 0.5 h. The reaction mixture was concentrated under reduced pressure to remove solvent. The residue was purified by prep-HPLC (column: Waters Xbridge BEH C18 100*30 mm*10um; mobile phase: [water (10 mM NH_4_HCO_3_)-ACN]; B%: 35%-65%, 8 min) to give **45** (5.9 mg, 9.90 µmol, 8.66% yield, 99.617% purity) as a white solid. MS: *m/z* 594.1 (M + H)^+^. ^1^H NMR (400 MHz, DMSO-d6) *δ* ppm 10.80 (s, 1H), 9.56 (s, 1H), 7.96 (d, 1H, *J =* 5.6 Hz), 7.92 (br t, 1H, *J =* 6.4 Hz), 7.44 (d, 1H, *J =* 8.0 Hz), 7.33–7.23 (m, 2 H), 7.06 (br d, 1H, *J =* 6.0 Hz), 6.98 (t, 1H, *J =* 7.6 Hz), 6.90 (d, 1H, *J =* 2.0 Hz), 6.85–6.78 (m, 1H), 6.60 (d, 2 H, *J =* 2.0 Hz), 6.57 (s, 1H), 3.74 (s, 3 H), 3.53 (br t, 1H, *J =* 3.6 Hz), 3.51–3.49 (m, 1H), 3.39 (br s, 1H), 3.09 (br dd, 1H, *J =* 5.2, 13.2 Hz), 2.56 (br s, 1H), 2.44 (br d, 1H, *J =* 14.0 Hz), 1.59 (br s, 2 H), 1.48 (s, 3 H), 1.42–1.24 (m, 2 H), 1.24–1.09 (m, 3 H), 1.40–1.07 (m, 1H).

##### *(S)-N-((1-(2*,*6-Difluoro-4-methoxyphenyl)cyclohexyl)methyl)-3-(1 H-indol-3-yl)-2-methyl-2-(3-(1-oxo-1*,*3-dihydroisobenzofuran-5-yl)ureido)propanamide* (**46**).

Compound **46** was obtained as a white solid using **68**[**46**] and 5-isocyanatoisobenzofuran-1(3 H)-one following the procedures to synthesize **45**. MS: m/z 631.1 (M + H)^+^. ^1^H NMR (400 MHz, DMSO-d6) *δ* ppm 10.80 (s, 1H), 9.41 (s, 1H), 7.96–7.87 (m, 2 H), 7.70 (d, 1H, *J =* 8.4 Hz), 7.46 (d, 1H, *J =* 8.0 Hz), 7.39 (d, 1H, *J =* 8.4 Hz), 7.28 (d, 1H, *J =* 8.0 Hz), 6.99 (t, 1H, *J =* 7.6 Hz), 6.91 (d, 1H, *J =* 1.6 Hz), 6.83 (t, 1H, *J =* 7.6 Hz), 6.63–6.53 (m, 3 H), 5.34 (s, 2 H), 3.74 (s, 3 H), 3.57–3.47 (m, 2 H), 3.40 (br d, 1H, *J =* 14.8 Hz), 3.10 (br dd, 1H, *J =* 5.2, 13.2 Hz), 2.50–2.33 (m, 2 H), 1.59 (br s, 2 H), 1.49 (s, 3 H), 1.38–1.09 (m, 6 H).

##### *(S)-3-(1 H-indol-3-yl)-N-((1-(5-methoxypyridin-2-yl)cyclohexyl)methyl)-2-methyl-2-(3-(1-oxo-2-phenylisoindolin-5-yl)ureido)propanamide* (**50**).

Compound **50** was obtained as a white solid using **55** and 5-isocyanato-2-phenylisoindolin-1-one following the procedures to synthesize **12**. MS: m/z 671.3 (M + H)^+^. ^1^H NMR (400 MHz, DMSO-d6) *δ* ppm 10.82 (s, 1H), 9.15 (s, 1H), 8.23–8.12 (m, 1H), 7.89 (br d, 3 H, *J =* 7.2 Hz), 7.63 (d, 1H, *J =* 8.4 Hz), 7.47–7.37 (m, 4 H), 7.33 (d, 1H, *J =* 8.8 Hz), 7.27 (d, 1H, *J =* 7.6 Hz), 7.20 (d, 1H, *J =* 8.8 Hz), 7.17–7.10 (m, 1H), 7.06–6.93 (m, 3 H), 6.83 (t, 1H, *J =* 7.6 Hz), 6.40 (s, 1H), 4.95 (s, 2 H), 3.64 (s, 3 H), 3.55–3.48 (m, 1H), 3.43 (br s, 1H), 3.39–3.38 (m, 2 H), 3.30–3.28 (m, 1H), 3.06 (br dd, 1H, *J =* 5.2, 12.8 Hz), 2.21–2.06 (m, 1H), 2.12 (br s, 2 H), 1.46 (br s, 4 H), 1.40 (s, 3 H), 1.38–1.31 (m, 1H), 1.24 (br d, 1H, *J =* 12.4 Hz), 1.10 (br s, 2 H).

##### *(S)-3-(1 H-indol-3-yl)-N-((1-(5-methoxypyridin-2-yl)cyclohexyl)methyl)-2-methyl-2-(3-((R)-1-oxo-3-phenyl-1*,*3-dihydroisobenzofuran-5-yl)ureido)propanamide* (**51**).

Compound **51** was obtained as a white solid following the procedures to synthesize **12**. MS: m/z 672.3 (M + H)^+^. ^1^H NMR (400 MHz, DMSO-d6) *δ* ppm 10.83 (d, 1H, *J =* 2.0 Hz), 9.12 (s, 1H), 8.17 (d, 1H, *J =* 3.2 Hz), 8.11 (d, 1H, *J =* 2.0 Hz), 7.53 (dd, 1H, *J =* 2.0, 8.4 Hz), 7.47–7.37 (m, 5 H), 7.35–7.27 (m, 4 H), 7.21 (d, 1H, *J =* 8.8 Hz), 7.04–6.96 (m, 3 H), 6.84 (t, 1H, *J =* 7.2 Hz), 6.66 (s, 1H), 6.34 (s, 1H), 3.63 (s, 3 H), 3.41 (br s, 2 H), 3.28–3.22 (m, 1H), 3.09 (br dd, 1H, *J =* 5.6, 13.2 Hz), 2.15 (br s, 2 H), 1.53–1.44 (m, 4 H), 1.42 (s, 3 H), 1.32–1.06 (m, 4 H).

##### *(S)-3-(1 H-indol-3-yl)-N-((1-(5-methoxypyridin-2-yl)cyclohexyl)methyl)-2-methyl-2-(3-((S)-1-oxo-3-phenyl-1*,*3-dihydroisobenzofuran-5-yl)ureido)propanamide* (**52**).

Compound **52** was obtained as a white solid following the procedures to synthesize **12**. MS: m/z 672.3 (M + H)^+^. ^1^H NMR (400 MHz, DMSO-d6) *δ* ppm 10.83 (s, 1H), 9.12 (s, 1H), 8.17 (d, 1H, *J =* 3.2 Hz), 8.12 (d, 1H, *J =* 2.0 Hz), 7.53 (dd, 1H, *J =* 2.0, 8.4 Hz), 7.47–7.38 (m, 5 H), 7.35–7.27 (m, 4 H), 7.21 (d, 1H, *J =* 8.8 Hz), 7.04–6.96 (m, 3 H), 6.84 (t, 1H, *J =* 7.2 Hz), 6.66 (s, 1H), 6.34 (s, 1H), 3.63 (s, 3 H), 3.37 (br d, 2 H, *J =* 6.0 Hz), 3.28–3.23 (m, 1H), 3.09 (br dd, 1H, *J =* 5.2, 13.2 Hz), 2.15 (br s, 2 H), 1.48 (br t, 4 H, *J =* 9.2 Hz), 1.42 (s, 3 H), 1.38–1.19 (m, 2 H), 1.14 (br d, 2 H, *J =* 10.0 Hz). 

##### *(S)-3-(1 H-indol-3-yl)-N-((1-(5-methoxypyridin-2-yl)cyclohexyl)methyl)-2-methyl-2-(3-((S)-3-oxo-1-phenyl-1*,*3-dihydroisobenzofuran-5-yl)ureido)propanamide* (**53**).

Compound **53** was obtained as a white solid following the procedures to synthesize **12**. MS: m/z 672.3 (M + H)^+^. ^1^H NMR (400 MHz, DMSO-d6) *δ* ppm 10.85 (s, 1H), 9.14 (s, 1H), 8.18 (d, 1H, *J =* 2.8 Hz), 8.13 (s, 1H), 7.54 (br d, 1H, *J =* 8.0 Hz), 7.48–7.40 (m, 5 H), 7.37–7.28 (m, 4 H), 7.22 (d, 1H, *J =* 8.8 Hz), 7.05–6.97 (m, 3 H), 6.85 (t, 1H, *J =* 7.2 Hz), 6.67 (s, 1H), 6.36 (s, 1H), 3.64 (s, 3 H), 3.40–3.38 (m, 2 H), 3.35–3.33 (m, 2 H), 2.16 (br s, 2 H), 1.48 (br s, 4 H), 1.43 (s, 3 H), 1.25 (s, 2 H), 1.14 (br s, 2 H).

*(S)-3-(1 H-indol-3-yl)-N-((1-(5-methoxypyridin-2-yl)cyclohexyl)methyl)-2-methyl-2-(3-((R)-3-oxo-1-phenyl-1*,*3-dihydroisobenzofuran-5-yl)ureido)propanamide* (**54**)

##### *(S)-3-(1 H-indol-3-yl)-N-((1-(5-methoxypyridin-2-yl)cyclohexyl)methyl)-2-methyl-2-(3-((R)-3-oxo-1-phenyl-1*,*3-dihydroisobenzofuran-5-yl)ureido)propanamide* (**54**).

Compound **54** was obtained as a white solid following the procedures to synthesize **12**. MS: m/z 672.3 (M + H)^+^. ^1^H NMR (400 MHz, DMSO-d6) *δ* ppm 10.83 (br s, 1H), 9.12 (s, 1H), 8.20–8.09 (m, 2 H), 7.53 (br d, 1H, *J =* 7.6 Hz), 7.47–7.37 (m, 5 H), 7.35–7.27 (m, 4 H), 7.21 (br d, 1H, *J =* 8.8 Hz), 7.04–6.94 (m, 3 H), 6.84 (br t, 1H, *J =* 7.6 Hz), 6.65 (s, 1H), 6.35 (s, 1H), 3.61 (s, 3 H), 3.43–3.38 (m, 2 H), 3.25 (br d, 1H, *J =* 6.4 Hz), 3.08 (br dd, 1H, *J =* 5.2, 12.8 Hz), 2.15 (br s, 2 H), 1.54–1.44 (m, 4 H), 1.41 (s, 3 H), 1.31–1.03 (m, 4 H).

## Supplementary Information

Below is the link to the electronic supplementary material.

Supplementary Material 1

## Data Availability

Data is provided within the manuscript or supplementary information files.
