# Supplementary material for: Discovery of a Potent Small Molecule Antagonist of GRPR for the Treatment of Pruritus
Source: Med Chem Res. 2026 May 26;35(6):1119–40. doi: 10.1007/s00044-026-03566-x (PMC13368858; doi:10.1007/s00044-026-03566-x)
Supplement: Supplementary file 1 — Supplementary Material 1 [file 44_2026_3566_MOESM1_ESM.docx]

**Discovery of a Potent Small Molecule Antagonist of GRPR for the Treatment of Pruritus**

Mingzhou Zhou^1,2^ • Roland E Dolle^1,2^ • Amruta Poreddy^3^ • Edmund Hudson^3^ • Michelle Schmidt^3^ • Margaret Grapperhaus^3^ • Tom Gordon^3^ • Huaping Chen^4^ • Mike Prinsen^1,2^ • Xianyu Liu^5,6^ • Zhiyong E. Tao^3^ • Jinbin Xu^4^ • Ma Xenia G Ilagan^1,2^ • Zhou-Feng Chen^5,6^

^1^ Center for Drug Discovery, Washington University School of Medicine, St. Louis, MO 63110, USA.

^2^ Department of Biochemistry and Molecular Biophysics, Washington University School of Medicine, St. Louis, MO 63110, USA.

^3^ Mallinckrodt Pharmaceuticals, Washington University School of Medicine, St. Louis, MO 63110, USA.

^4^ Mallinckrodt Institution of Radiology, Washington University School of Medicine, St. Louis, MO 63110, USA.

^5^ Center for the Study of Itch & Sensory Disorders, Washington University School of Medicine, St. Louis, MO 63110, USA.

^6^ Department of Anesthesiology, Washington University School of Medicine, St. Louis, MO 63110, USA.

**Material and methods for radiobinding assay:**

Data from the competitive binding experiments were modeled using nonlinear regression analysis to determine the inhibitor concentration that inhibits 50% of radioligand-specific binding (*IC*_50_). The competition curves were fitted to a single-site binding model using the following equation:

| $B_{s}=B_{0}-\left[\frac{\left(B_{0}*I\right)}{\left(IC_{50}+I\right)}\right]$ | (1) |
| --- | --- |

Where, *B*_s_ is the amount of the radioligand bound specifically to the cells (i.e., *B*_s_ = *B*_t_ – *B*_ns_, where *B*_t_ is the total bound radioactivity and *B*_ns_ is the nonspecific binding of the radiotracer), *B*_0_ is the amount of the radioligand bound in the absence of the competitive inhibitor, *I* is the concentration of the competitive inhibitor and the *IC*_50_ is the concentration of the competitive inhibitor that blocks 50% of the total specific binding, or 50% sites occupied by the competitor. Data from competitive radioligand binding studies were transformed to determine the pseudo-Hill coefficient, *n’*_H_, defined as:

| $\log\frac{B_{s}}{B_{0}-B_{s}}=n\text{'}_{H}\log I-n\text{'}_{H}\log IC_{50}$ | (2) |
| --- | --- |

*n’*_H_, which is the negative of the Hill slope, was readily determined from the plot of $\log\frac{B_{s}}{B_{0}-B_{s}}$ versus $\log I$.

**A B C**

**Fig. S1** Competitive binding for inhibition of the ^125^I-Tyr4-Bn binding to MTH1 in GRPR cell homogenates by PD 176252 and MP-4222. A: Representative competitive binding data IC50 for PD 176252: 91.75 ± 19.34 nM and MP-4222: 21.15 ± 2.82 nM. B (PD176252) and C (MP-4222): Representative PseudoHill plots for determining the PseudoHill coefficient (n’H values). PD 126252: n’H = 0.84 ± 0.2; MP-4222: n’H = 0.40 ± 0.05. n = 3, samples in triplicate, mean ± SE.


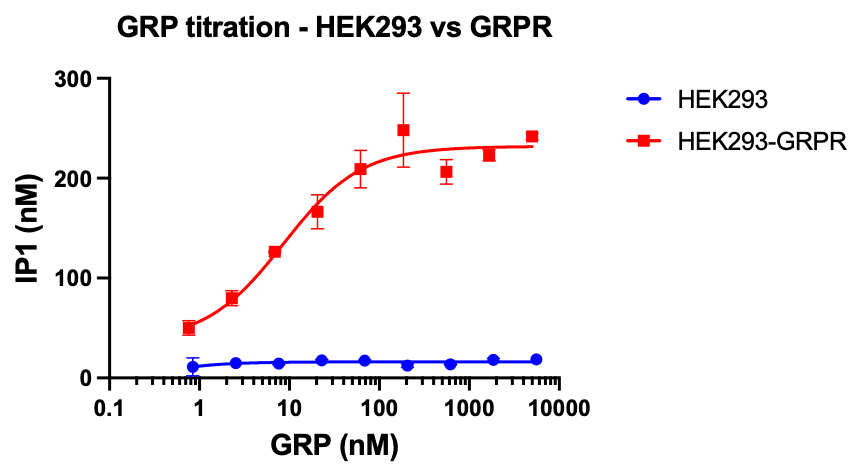


**Fig. S2** GRP dose-response curve in GRPR stable and parental cell lines


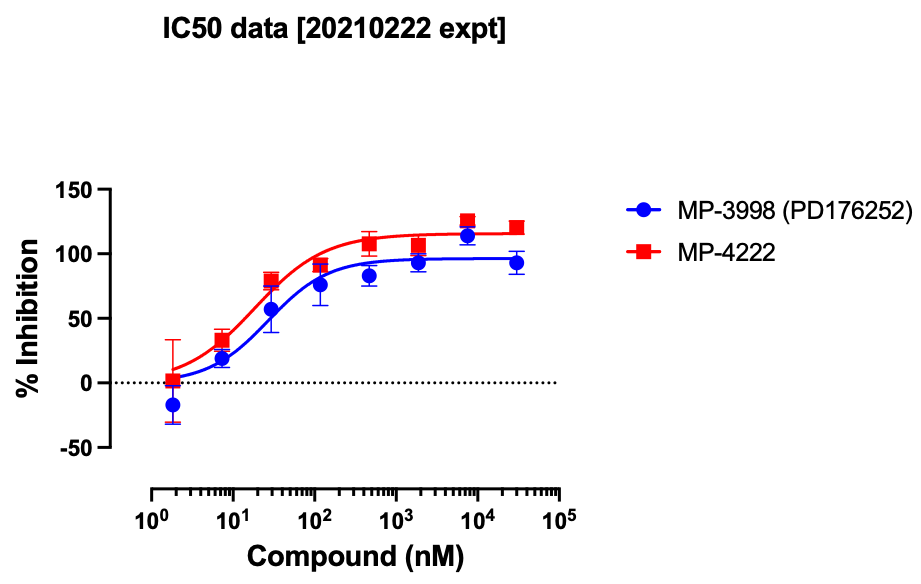


**Fig. S3** Inhibition curves for MP-4222 (**45**) and MP-3998 (PD 176252)

**Characterization of 45**:
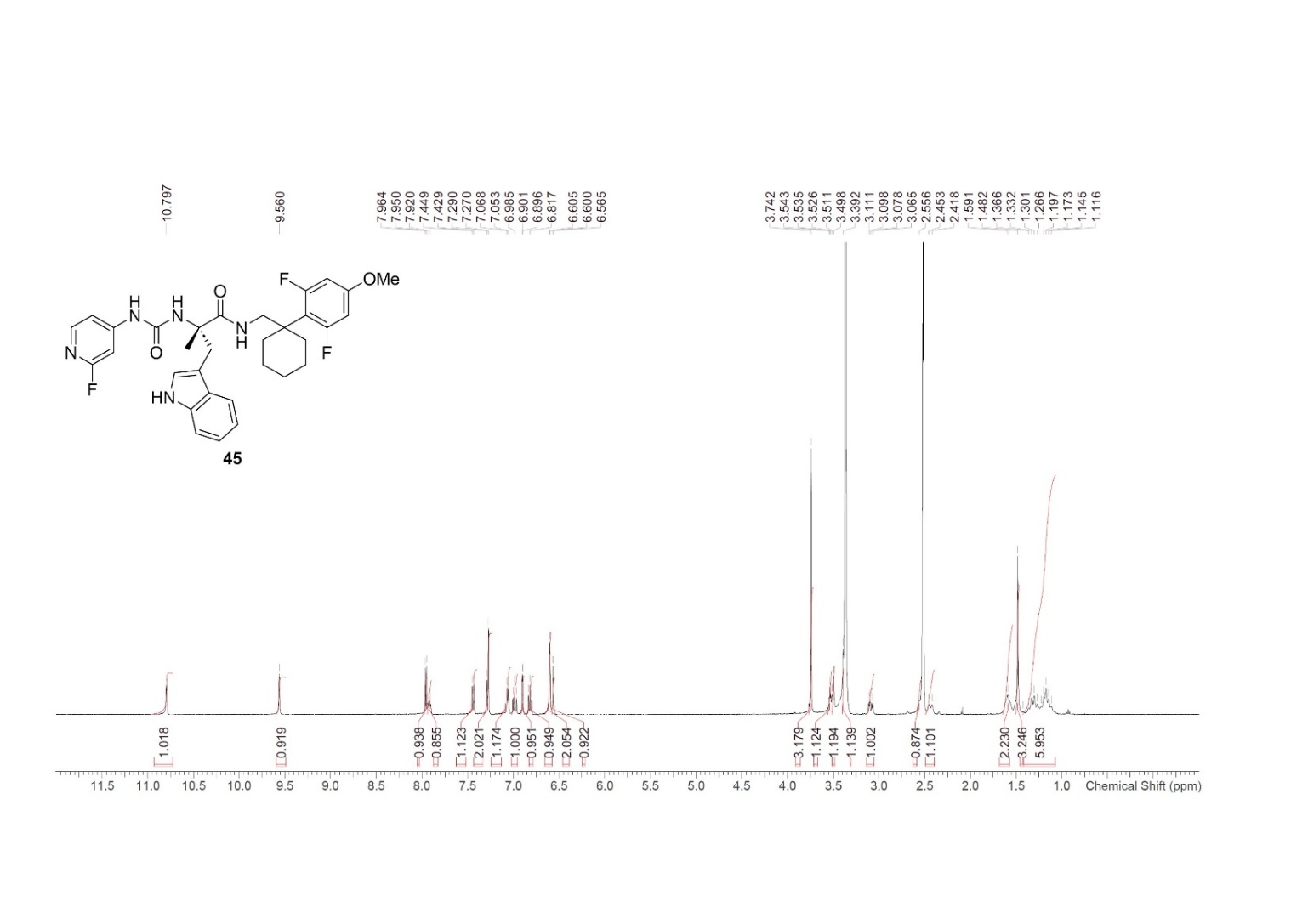


**Fig. S4** 1HNMR of **45**


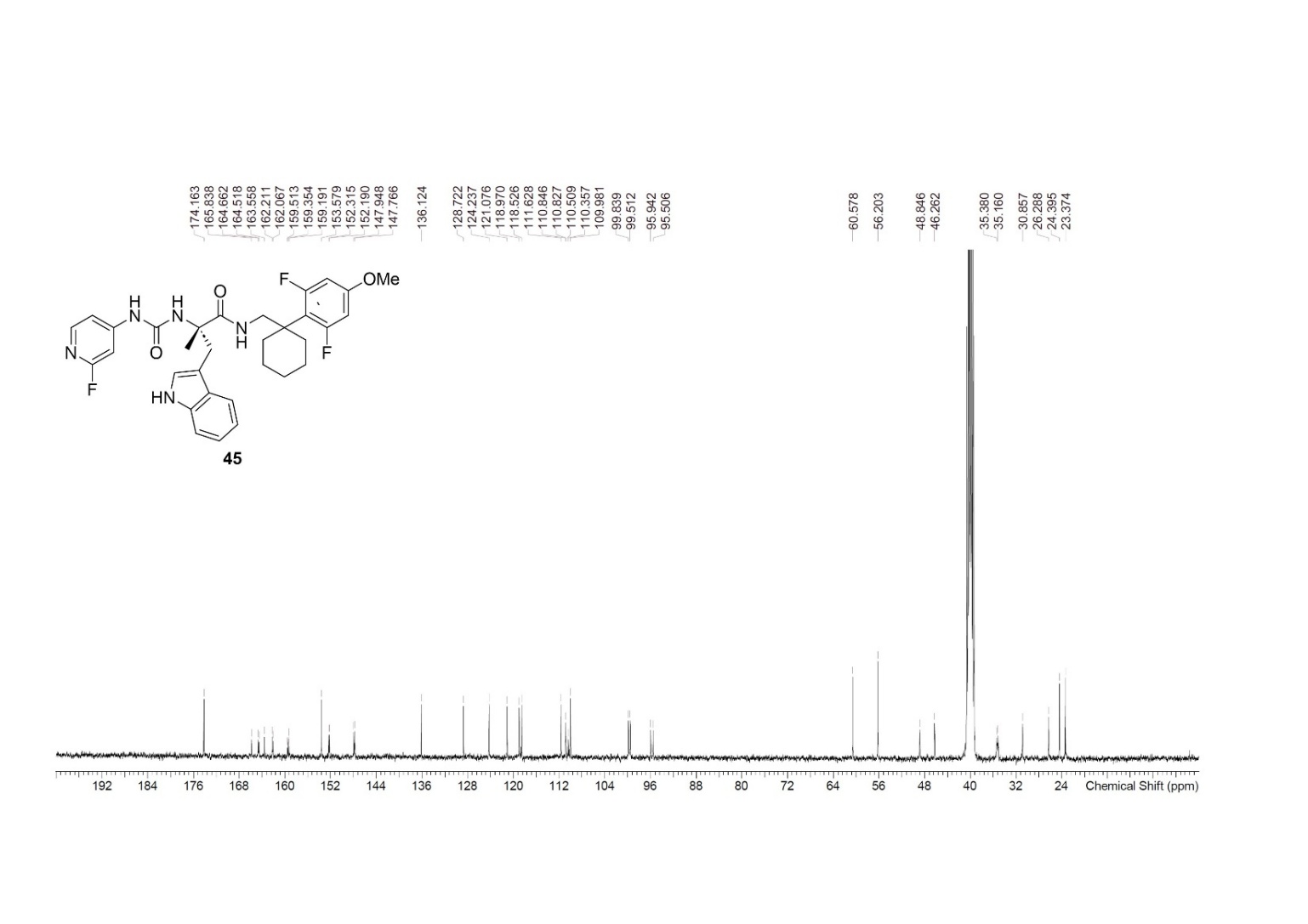


**Fig. S5** 13CHNMR of **45**


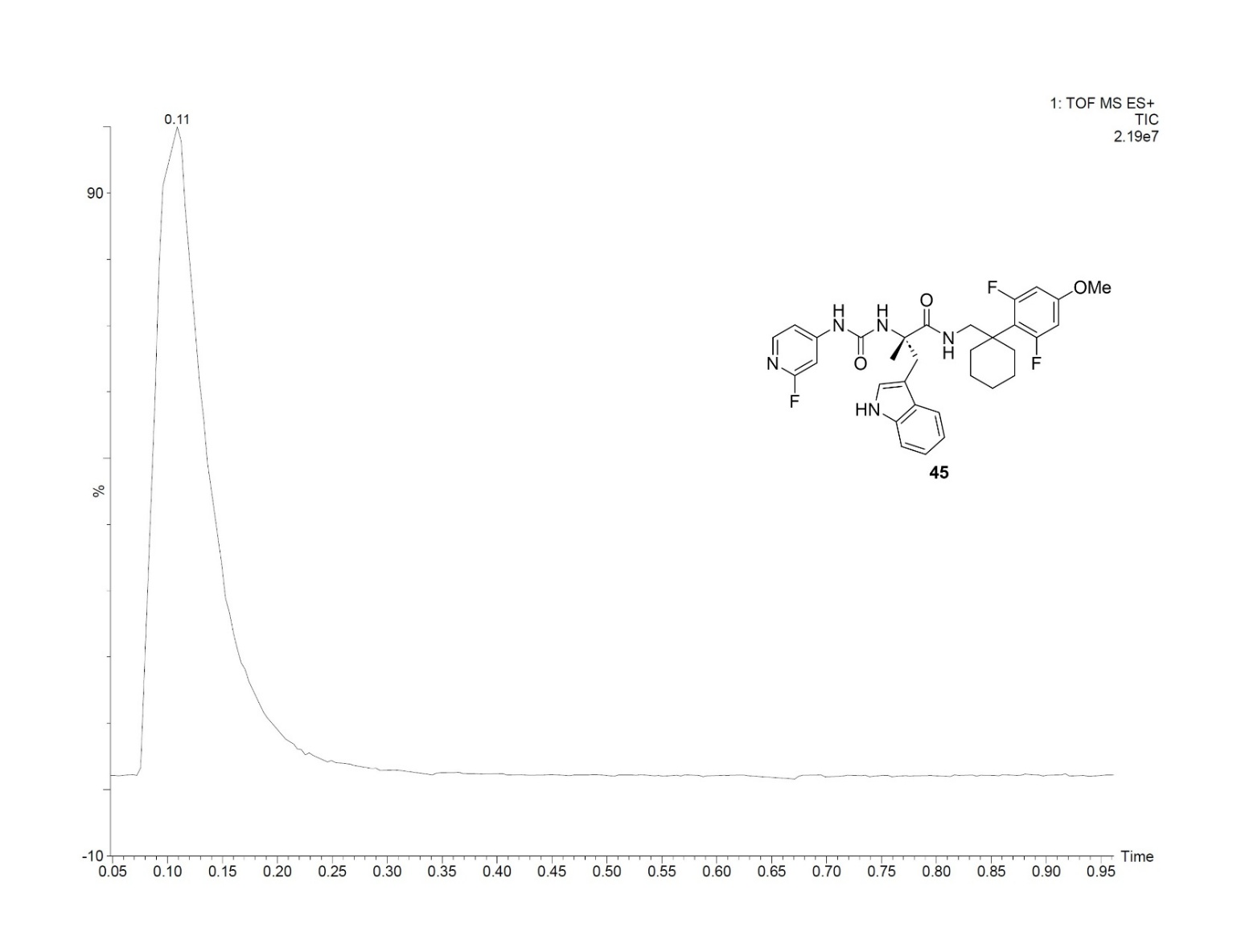

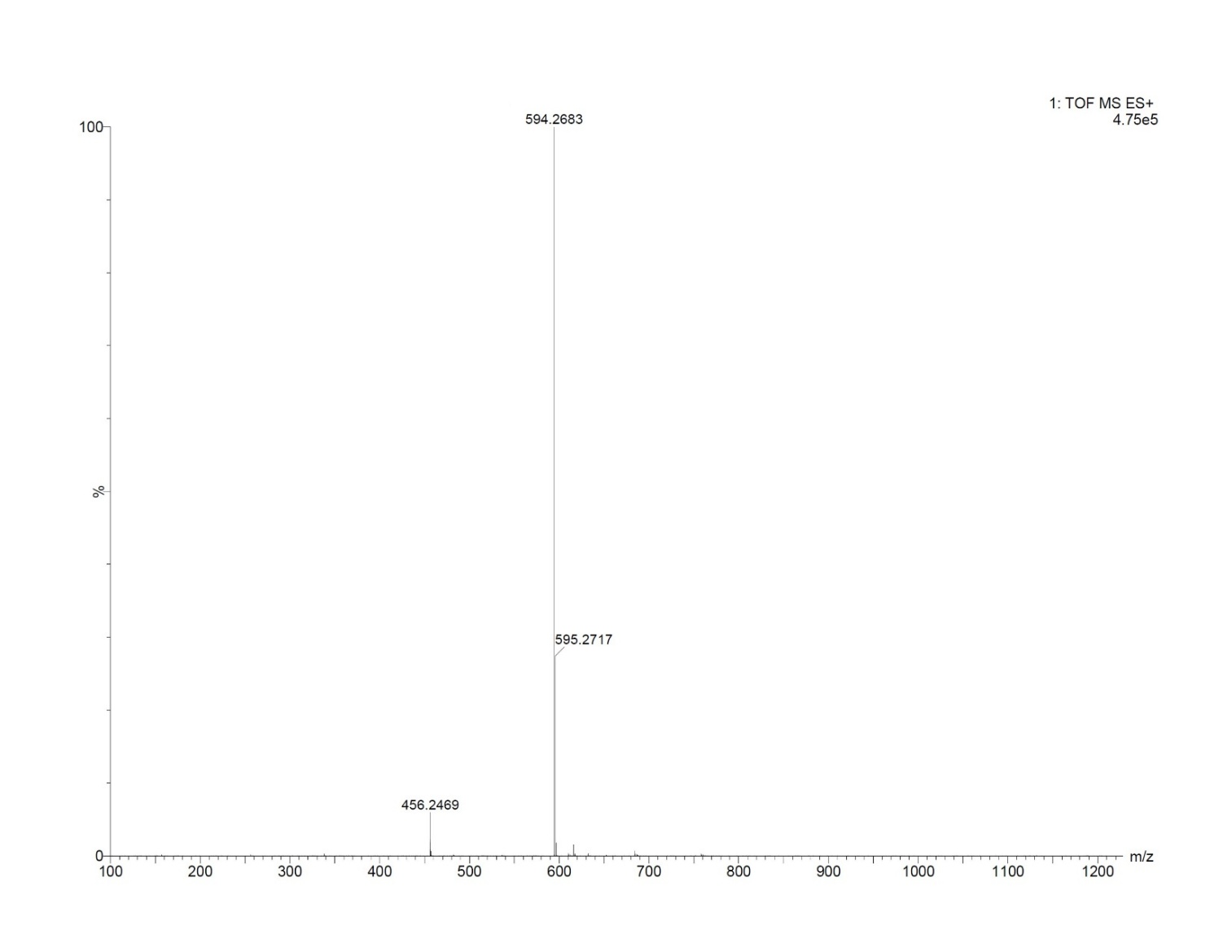


**Fig. S6** HRMS of **45**
